# Methods based on a semi-empirical model for simulating scroll compressors with HFC and HFO refrigerants

**DOI:** 10.12688/openreseurope.14313.2

**Published:** 2022-03-25

**Authors:** George Meramveliotakis, George Kosmadakis, Sotirios Karellas

**Affiliations:** 1 Thermal Hydraulics and Multiphase Flow Laboratory, Institute of Nuclear & Radiology Sciences & Technology, Energy & Safety, National Center for Scientific Research Demokritos, Agia Paraskevi, 15341, Greece; 2 Laboratory of Steam Boilers and Thermal Plants, School of Mechanical Engineering, National Technical University of Athens, Athens, 15780, Greece

**Keywords:** scroll compressor, refrigerants, semi-empirical model, heat transfer, pressure drop, leakages

## Abstract

The aim of this work is to evaluate three methodologies regarding semi-empirical scroll compressor modeling for different refrigerants and conduct a comparative analysis of their results and accuracy. The first step is to improve a semi-empirical model for scroll compressors based on established techniques, and further enhance the physical background of some of its sub-processes leading to more accurate predictions. Focus is then given on the compressor operation when changing the refrigerant, proposing three methods in total. The first method refers to the standard model, requiring an optimization process for the calibration of all the model parameters. The second method relies on a reference refrigerant, and also uses optimization procedures, but for the fine-tuning of a small subset of the parameters. The third method is more generalized, without the need of any optimization process for the parameters identification, when fluid change occurs, leading to a very fast approach. Το evaluate the accuracy and verify the applicability of each method also related to the necessary computational time, two scroll compressors each with three different refrigerants are considered (HFCs and HFOs and their blends). The model is evaluated with the available manufacturer data, using R134a as reference refrigerant. The results show that the first method predicts the key indicators with a very high accuracy, with the maximum discrepancy of 2.06%, 4.17% and 3.18 K for the mass flow rate, electric power and discharge temperature respectively. The accuracy of the other two methods is dropping, but within acceptable levels in most of the cases. Therefore, in cases that reduced accuracy can be accepted, the third method is preferred for compressor performance prediction when changing the refrigerant, which provides results at a small fraction of time compared with the other two methods, once the parameters are calibrated for a reference case.

## Nomenclature

**Table:** 

A	Area of heat exchanger	m ^2^
corr	Slip coefficient	-
c _p_	Specific heat capacity	kJ·kg ^-1^·K ^-1^
*Ċ*	Heat capacity	kW·K ^-1^
d	Diameter	m
h	Enthalpy	kJ·kg ^-1^
K	Friction factor	1/m ^4^
L	Characteristic length	m
M	Mach number	-
*ṁ*	Mass flow rate	kg·s ^-1^
N	Compressor speed	s ^-1^
n	Efficiency	-
Nu	Nusselt number	-
P	Pressure	kPa
Pr	Prandtl number	-
$\dot{Q}$	Thermal capacity	kW
Re	Reynolds number	-
r _v_	Build-in volume ratio	-
s	Specific entropy	kJ·kg ^-1^·K ^-1^
T	Temperature	°C
U	Overall heat transfer coefficient	kW·m ^-2^·K ^-1^
u	Velocity	m·s ^-1^
v	Specific volume	m ^3^·kg ^-1^
${\dot{V}}_{s}$	Volumetric flow rate	m ^3^·s ^-1^
V	Volume	m ^3^
w	Specific work	kJ·kg ^-1^
*Ẇ*	Compressor electrical power	kW
*Z*	Number of data sets	-

## Greek letters

**Table:** 

α	Compressor losses variable term	-
γ	Isentropic exponent	-
Δ	Difference	-
ε	Heat transfer effectiveness	-
Θ	Optimization function	-
λ	Thermal conductivity	kW·m-1·K ^-1^
μ	Dynamic viscosity	Pa·s
ξ	Drag factor	-
ρ	Density	kg·m ^-3^
σ	Standard deviation	-

## Subscripts

**Table:** 

0	Initial value
ad	Adapted conditions
amb	Ambient
calc	Calculated value
cp	Compressor
crit	Critical condition
data	Data obtained from manufacturer
ex	Exhaust
in	Internal
is	Isentropic
leak	Leakage
loss	Compressor losses
n	Nominal
r	Refrigerant
ref	Reference
rel	Relative
s	Swept
su	Suction
thr	Nozzle throat
v	Isochoric
vol	Volumetric
w	Wall

## Abbreviations

**Table:** 

ANN	Artificial neural network
COP	Coefficient of performance
GHG	Greenhouse gas
GWP	Global warming potential
HC	Hydrocarbon
HCFC	Hydrochlorofluorocarbon
HCFO	Hydrochlorofluoroolefin
HFC	Hydrofluorocarbon
HFO	Hydrofluoroolefin
HTHP	High temperature heat pump
ODP	Ozone depletion potential

## Introduction

Refrigeration and heat pump systems are widely used nowadays in a variety of applications, from industrial to residential ones. The effort during the last decades is to reduce the ozone depletion potential (ODP) and greenhouse gas emissions (GHGs) related to their use and the charged refrigerant. For that reason, many regulations and policies have come into force already since the 1980s with the Montreal Protocol, by controlling refrigerants that deplete the ozone layer like chlorofluorocarbons (CFCs) and hydrochlorofluorocarbons (HCFCs). Hydrofluorocarbons (HFCs) are more ozone-friendly refrigerants and have initially replaced those substances. But still HFCs have a high global warming potential (GWP), usually higher than 1000. In order to combat this effect aiming to limit and phase out refrigerants with high GWP in refrigeration applications, other policies have been introduced such as the EU F-Gas regulation ^1^ and Paris Agreement (2015). According to Kigali amendment to the Montreal Protocol ^2^, the HFCs are going to be reduced in terms of production and consumption, emerging the need for development, evaluation and implementation of new low GWP (GWP<150) refrigerants. Based on these requirements, many efforts have been done to find alternatives, such as natural refrigerants, hydrocarbons (HCs), hydrofluoroolefins (HFOs) and hydrochlorofluoroolefins (HCFOs) ^3^. Attention is also given on refrigerant mixtures ( *e.g.* HFC with HFO blends) due to their wide flexibility in thermodynamic and GWP characteristics, since pure substances are not so easily adapted. Future refrigerants need to provide a combination of acceptable performance in refrigeration systems, zero or almost zero ODP and very low GWP, in order to be considered as potential alternatives in the future.

The evaluation of the performance of such refrigerants in existing refrigeration and heat pump applications is not an easy task due to the lack of sufficient data, especially related to the compressor operation. In order to tackle this, both experimental and computational campaigns have been initiated, relying on existing compressors, attempting to identify the potential of each new refrigerant or mixture.

As experimental evaluation is concerned, many researchers have conducted drop-in experiments, in order to estimate the performance of low GWP refrigerants and mixtures in heat pump applications. Babiloni *et al.* ^4^ studied experimentally the HFO R1234ze(E) and its mixture R515B as replacements of R134a in a heat pump water heater with a scroll compressor. The results including 65 experimental datasets revealed that the heating capacity with R1234ze(E) and R515B is reduced by 25% and 27% respectively, compared with R134a due to the decreased mass flow rates. On the other hand, a slightly higher compressor performance was observed with these alternative refrigerants, improving the coefficient of performance (COP) by about 5% at high condensation temperatures. Fukuda *et al.* ^5^ performed a numerical and experimental evaluation with R1234ze(E) and R1234ze(Z) for high supply temperatures using an existing hermetic twin rotary compressor developed for R410A. Their results showed that R1234ze(Z) enhances the COP at condensation temperatures of 105 and 125 °C, proposing that the low GWP refrigerants R1234ze(E) and R1234ze(Z) could be used as alternatives to high temperature heat pump (HTHP) systems.

Another drop-in experiment was conducted by Thu *et al.* ^6^ using a low GWP mixture composed by R32/R1234yf/R744. They focused on the energy and exergy performance of domestic heat pump applications. For that reason, one cooling and two heating modes have been evaluated in various loads, with the cycle exergetic efficiencies being 31.7%, 37.8% and 43.3% respectively. Moreover, Sanchez *et al.* ^7^ tested a hermetic reciprocating compressor with two HFOs, R1234yf and R1234ze(E), under a wide operating range. Their aim was to identify whether these two ultra-low GWP refrigerants can be drop-in alternatives to R134a. Their results revealed that R1234yf decreased the cooling capacity by 4.5-8.6% and increased the power consumption by 1.6-6.7%, leading to a COP reduction of about 10%. The tests with R1234ze(E) also showed a lower cooling capacity by 24.9%, but a lower power consumption of 17.8% compared with R134a, leading to a COP reduction of about 8.6%.

As the main component of a heat pump or refrigeration system is the compressor, whose performance greatly affects the overall system performance ^8^, many methods have focused on modeling this component with high detail, relying on various numerical techniques. Aiming to develop a multi-purpose numerical model suitable for a wide range of positive displacement machines including both compressors and expanders Bell *et al.* ^9^ and Ziviani *et al.* ^10^ presented a generalized algorithm examining four types of compressors (hermetic reciprocating compressor, hermetic rolling-piston compressor, open-drive z-compressor and open-drive single screw compressor). Moreover, Ma *et al.* ^11^ using artificial neural networks (ANN) developed a generalized model for compressor simulation validating this model with three available compressors.

On the other hand, focusing only on reciprocating compressors, Duarte *et al.* ^12^ and Navarro *et al.* ^13^ presented numerical models for steady state modeling while Yang *et al.* ^14^ developed a comprehensive model taking into consideration compression process, geometry and kinematics, valves and leakages, heat transfer and frictional power losses sub-models. Ndiaye and Bernier ^15^ extended their model for unsteady/ dynamic conditions for simulating the on-off operation on hermetic reciprocating compressors. The model was validated for both transient and steady-state conditions of a water to air heat pump with R22 in both heating and cooling modes.

Other researchers have focused on implementing numerical models in order to simulate compressor and overall system performance operating with different refrigerants. For that reason, the so-called semi-empirical models can be used, demanding less information than the detailed compressor models. Due to their simplicity, semi-empirical models can be easily adjusted to simulate compressor performance for different operating conditions and working fluids. Byrne *et al.* ^16^ developed a simplified model for scroll compressor validating it for R407C using experimental data. Then, the model was generalized based on the methodology proposed by Duprez *et al.* ^17^ and adapted to hydrocarbons R290, R1270 and R600a. A numerical comparison was carried out, showing that the mass flow rate was expected to be lower for the alternative refrigerants, negatively affecting the system capacity. Furthermore, the electric power was estimated to vary by -12.5%, +6.8% and -63.9% for R290, R1270 and R600a respectively, compared with R407C.

Another semi-empirical assessment has been done by Royo *et al.* ^18^ investigating the replacement of R245fa with the low-GWP R1224yd(Z) refrigerant (an HCFO) in a HTHP configuration. Initially, based on an experimental setup, performance data with R245fa wereobtained, and then the system performance with R1224yd(Z) was predicted through semi-empirical calculations. Their methodology consisted of a number of assumptions for the heat pump cycle (e.g. constant sub-cooling, superheat) and variable isentropic and volumetric efficiency calculated from experimental results. The overall conclusion is that the alternative working fluid brings a lower power consumption by 7–11%, while the heating capacity is reduced between 7.2% and 8.9% compared with the R245fa. Additionally, Kosmadakis *et al.* ^8^ examined numerically the performance and cost effectiveness of different HTHP cycle designs with screw compressors, for three low GWP refrigerants, R1234ze(Z), R1233zd(E) and R1336mzz(Z). The simulation model solves numerically the overall heat pump cycle and correlations of the isentropic and volumetric efficiencies have been developed as a function of volume flow rates based on performance data obtained from the manufacturer, easily extrapolated to other refrigerants.

A similar approach has been followed for simulating expanders (compressors in reverse), such as by Muye *et al.* ^19^, who developed a general semi-empirical model for scroll expanders working with R134a. The model was then adjusted for different working fluids and validated using experimental data for ammonia/water mixture and pure ammonia as working fluids. The accuracy was then 5%, 7% and 4 K for mass flow, electric power and discharge temperature respectively, verifying the use of the generalized model for modeling the expander operation with different refrigerants. Table 1 provides an overview of all models highlighted above, both the ones focusing on the detailed compressor modeling but also the ones extending their applicability for various refrigerants. 

**Table 1.** Overview of compressor models.

Model overview	Machine type	Accuracy	Refrigerants	Reference
Mechanistic-deterministic for simulating quasi steady performance	Compressor/ expander	Mean average percentage error < 9.9% for electricity, <6.1% for mass flow rate and < 2% for dischage temperature	R410A, R245fa, SES36	(Bell *et al.* ^9^, Ziviani *et al.* ^10^)
Automated compressor mapping model using neural networks	Compressor	Mean average percentage error < 3.1% for electricity and <2.1% for mass flow rate	R410A, R407C	(Ma *et al.* ^11^)
Phenomenological model for steady state performance	Reciprocating compressor	Mean relative difference -0.2% for discharge temperature and 2.6% for mass flow	R134a	(Duarte *et al.* ^12^)
Phenomenological model based on refrigerant ideal evolution	Hermetic reciprocating compressor	Lower than 5% compared with test data	Propane	(Navarro *et al.* ^13^)
Comprehensive numerical model including three main sub models	Semi-hermetic reciprocating compressor	Mean absolute error 4.03% for mass flow rate and 6.43% for electric power	CO _2_	(Yang *et al.* ^14^)
Numerical model simulating both steady state and transient conditions	Hermetic reciprocating compressor	Heating mode: mass flow rate underpredicted by 7.4% and power underpredicted by 2.2% Cooling mode: mass flow rate underpredicted by 8.5% and power overpredicted by 3.6%	R22	(Ndiaye & Bernier, ^15^)
Semi empirical model calibrated with experimental data in order to investigate operation with different refrigerants	Scroll compressor	-	R1224yd(Z), R245fa, R407C, R290, R1270, R600a	(Byrne *et al.* ^16^, Duprez *et al.* ^17^, Mateu-Royo *et al.* ^18^)
Semi empirical for scroll expander modeling with different working fluids	Scroll expander	Less than 5%, 7% and 4K for mass flow rate, shaft power and exhaust temperature	R134a, ammonia-water, pure ammonia	(Muye *et al.* ^19^)

Among the different numerical approaches, the semi-empirical model is perhaps the more reliable for volumetric machines of various types (scroll, reciprocating, screw), since it includes several sub-processes and once its parameters are fine-tuned, its prediction accuracy can be very high. However, this fine-tuning relies on the use of experimental or manufacturer data, which might not be available, especially when HFOs or their blends are considered. In this work, a semi-empirical model for scroll compressor is developed predicting the mass flow rate, electric power and discharge temperature, according to established methodologies ^20– 22^. The aim is to provide insights about the prediction accuracy and results reliability when the semi-empirical model is used, in order to evaluate the performance of a scroll compressor, when changing the refrigerant. For that reason, three methodologies are proposed, in order to simplify the necessary parameters and generalize the model for different refrigerants. The first two methods require the availability of experimental or manufacturer data, in order to calibrate the model parameters, while the third method generalizes the model for any fluid operation without the need of any data since the model is calibrated only once for the reference refrigerant. The comparison of the model results with the available data provides the accuracy of the three methods, when using different compressors and refrigerants. The outcome of this study is to enlighten the semi-empirical model accuracy with the use of the three suggested methods especially when performance data is not a priori available, with the final aim to develop a reliable tool towards the better evaluation of alternative refrigerants in heat pump systems.

## Methodologies and semi-empirical modeling for scroll compressor

The semi-empirical model of a scroll compressor developed by Winandy *et al.* ^20^ is the starting point of the methodology proposed in the current work. Based on this approach, further sub-processes have been added to the original model enhancing its physical background and improving its prediction accuracy ^23^. These adjustments have led to different versions/methods and are evaluated in a wide range of operating conditions obtained from the manufacturer’s data, as well as two compressors and different refrigerants.

Three methodologies regarding the identification of the model parameters are proposed and their results compared, aiming to keep a high prediction accuracy and at the same time to reduce the computational time needed, when changing the refrigerant. This becomes crucial when there are no data available for a compressor-refrigerant pair. The three methods are highlighted next:

1. The first method refers to the standard model using an optimization process for the calibration of all the model parameters, requiring the availability of performance data for the refrigerant of concern.
2. The second one sets a reference refrigerant for which data are available, and the refrigerant change requires the fine-tuning of a small subset of the total number of parameters (the refrigerant-specific ones), but still requiring performance data to be available.
3. The third method is further simplified without the need of any optimization process once changing the refrigerant, with the refrigerant-specific parameters calculated by standard correlations based on a reference refrigerant, with the other parameters kept constant (the compressor-specific ones). This method has the advantage that the semi-empirical model can be calibrated without knowing any performance data for the specific refrigerant.

The next sections describe first the complete adapted semi-empirical model, which actually concerns the first method highlighted before once the full set of parameters is included. The second and third method also rely on the same semi-empirical model, once the number of parameters to be optimized are reduced (method 2) or eliminated (method 3).

### Description of the adapted semi-empirical model

The original semi-empirical model ^20^ considers four steps to describe the process of the vapour refrigerant inside the compressor and its goal is to predict the following key indicators: the exhaust temperature, the mass flow rate, and the electrical power. The necessary inputs are the supply temperature, the evaporating and condensing pressures, the ambient temperature, and the compressor speed.

**Figure 1.**
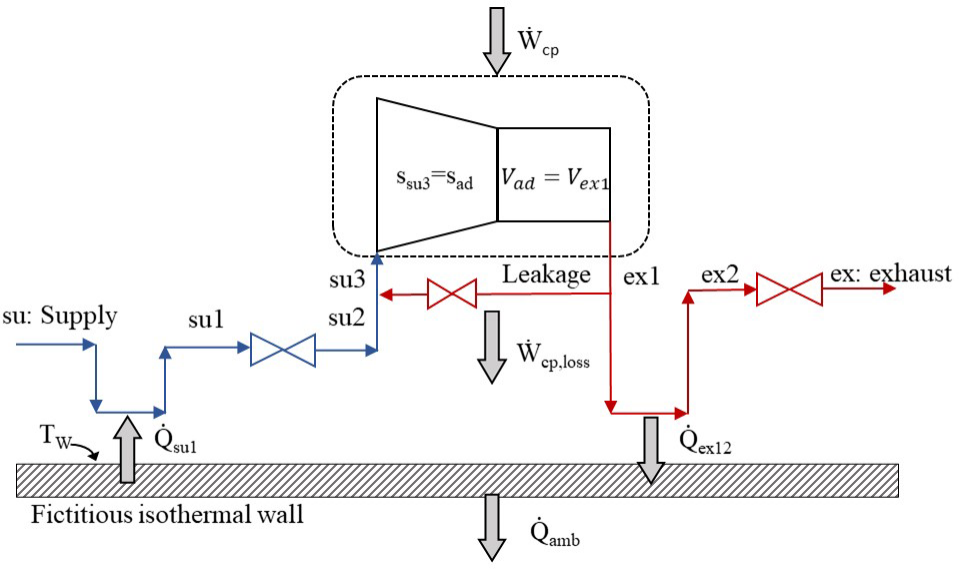
The refrigerant sub-processes within the compressor.

Further steps are introduced here, with some inspired by D’Amico *et al.* ^23^, in order to enhance the physical background of the process also including the pressure drop at the suction and exhaust ports and leaking refrigerant within the compressor. The performance indicators have been defined based on recent practices ^24^ as a function of a set of parameters with physical importance that can be determined from experimental or manufacturer data. The evolution of the refrigerant through the compressor is shown in Figure 1 and consists of eight steps in total.

**Figure 2.**
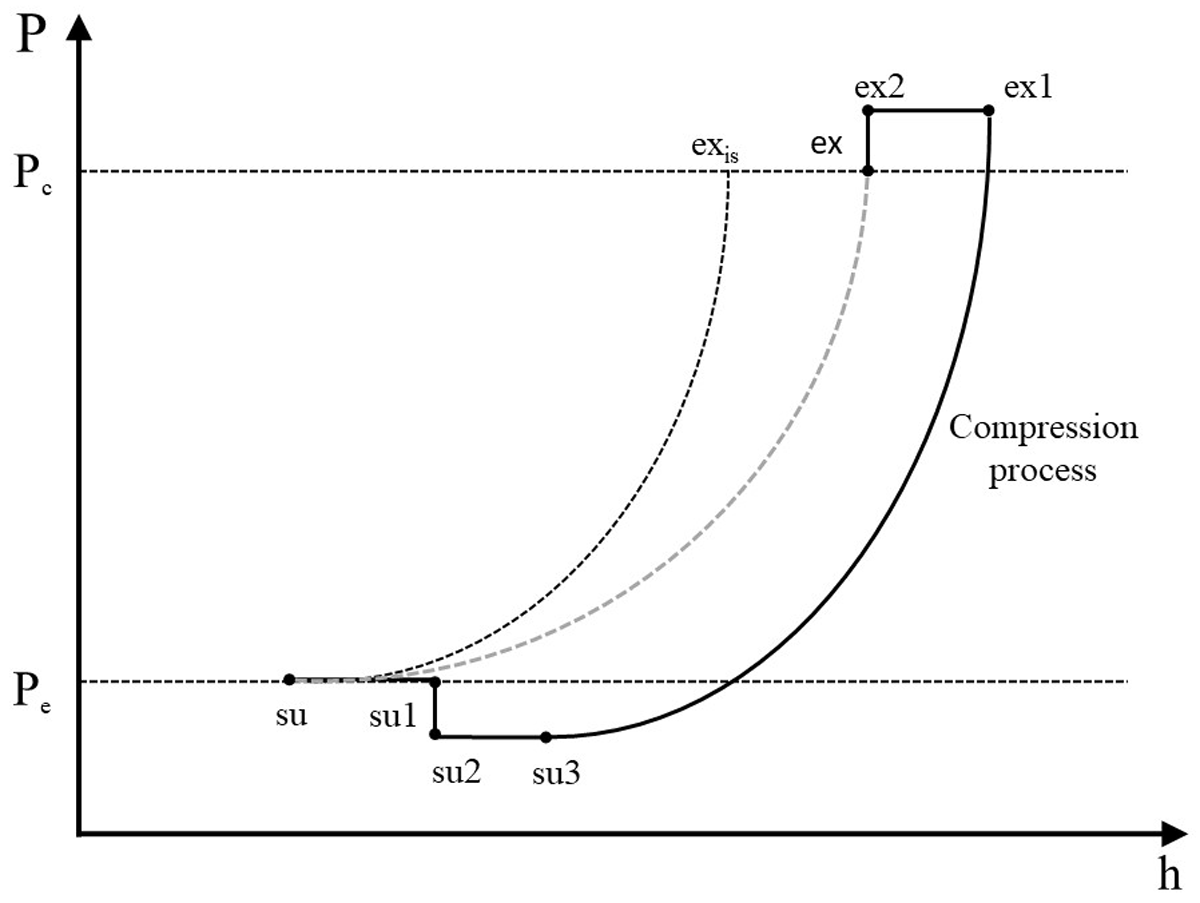
Refrigerant processes during compression in a pressure-enthalpy diagram.

The refrigerant enters the compressor at state 1 (su) and exits at state 8 (ex) with the sub-processes described below and illustrated in the pressure-enthalpy chart of Figure 2.

1. Isobaric heating-up, due to the hot motor and the casing, which are maintained at a higher average temperature than the refrigerant’s suction temperature (su→su1).
2. Supply (adiabatic) pressure drop at the suction port and the motor (su1→su2).
3. Mixing of supply flow with the leakage flow, with the latter undergoing a throttling process which reduces its pressure (su2→su3).
4. Isentropic compression of the refrigerant to the intermediate pressure (su3→ad).
5. Adiabatic compression from the intermediate pressure to the exhaust one (before pressure drop) at constant volume (ad→ex1).
6. Leaking flow to the compressor inlet (ex1→supply line).
7. Exhaust isobaric cooling due to the lower temperature of the fictitious isothermal wall than the exhaust refrigerant (ex1→ex2).
8. Pressure drop at the exhaust port reaching the discharge pressure (ex2→ex3).

***Description of the refrigerant processes during compression.*** Scroll compressors are volumetric machines and they are characterized by a fixed build-in volume ratio, *r _v,in_*, which results to a predefined refrigerant compression up to a certain volume ^22^. For a given refrigerant and operating conditions, there is a unique pressure ratio that corresponds to this volume ratio. Ideally, the compressor should be operated under this pressure ratio, but this is not possible in most cases, because the external (operating) pressure ratio is different than the internal one, leading to either over-compression or under-compression requiring additional power for the process. This diversion from the ideal compression is modeled by splitting the process into two steps ^20^. The first step of the compression is an isentropic process from the suction pressure to the internal (adapted) pressure, which is directly related to the build-in volume ratio. The second part is an adiabatic compression at constant volume (but not reversible) giving either a negative work in case of over-compression or a positive work in case of under-compression. This second part corresponds to the opening of the compression chamber to the discharge plenum. The total work of these two processes is given by Equation (1).


$$w_{in}=w_{in,is}+w_{in,v}=(h_{r,ad}-h_{r,su3})+v_{r,in}\cdot (P_{ex1}-P_{ad})\;(1)$$

where *w _in_* is the total work provided to the compressor, *w _in,is_* is the required work for the isentropic compression from the initial to the build-in volume, and *w _in,v_* is the work corresponding to the constant volume part.

The mass flow rate ( *ṁ _r,cp_* ) is calculated by Equation (2), where *ρ*_*su*3_ is the density after suction heating up and pressure drop, *V _s_* is the swept volume of the compressor (model parameter) and *N _cp_* is the compressor speed.


$${\dot{m}}_{r,cp}=\rho_{su3}\cdot V_{s}\cdot N_{cp}\;(2)$$

This flow rate includes a part due to leakages ( *ṁ _r,cp_*), as shown in Equation (3).


$${\dot{m}}_{r,cp}={\dot{m}}_{r}+{\dot{m}}_{r,leak}\;(3)$$

Introducing the motor slip coefficient, the actual compressor speed *N _cp_* ^22^ is calculated by Equation (4).


$$N_{cp}=N_{n,cp}\cdot (1-corr\cdot {\dot{W}}_{cp})\;(4)$$

where *Ν _n,cp_* is the nominal compressor rotational speed, *corr* is the slip coefficient, and *Ẇ _cp_* is the actual compressor power.

The compressor electrical power includes two terms, the first is the internal compression power, *Ẇ _in,cp_*, and the second is the electromechanical losses, *Ẇ _cp,loss_*. Τhe last is split into two terms ( Equation (5)), which express the constant losses, *Ẇ*_*cp,loss,*0_, and the variable losses that are proportional to the internal compression power ( *Ẇ _cp,in_*).


$${\dot{W}}_{cp}={\dot{W}}_{cp,in}+{\dot{W}}_{cp,loss}={\dot{W}}_{cp,in}+{\dot{W}}_{cp,loss,0}+a_{cp}\cdot {\dot{W}}_{cp,in}\;(5)$$

Heat transfer

The model considers the refrigerant (isobaric) heating-up, because of the hot compressor casing at the suction, ${\dot{Q}}_{su1}$ , and exhaust (isobaric) cooling down, ${\dot{Q}}_{ex12}$ , due to the higher refrigerant temperature at the discharge. The model neglects the heat transfer between refrigerant and oil. These two heat transfer processes are modeled with the NTU method. For the heating up process Equation (6)– Equation (9) are used and with the same approach also adjusted for the discharge cooling down.


$${\dot{C}}_{su}={\dot{m}}_{r}\cdot c_{p,su}\;(6)$$

$${\dot{Q}}_{su1}={\dot{C}}_{su}\cdot (T_{su1}-T_{su})\;(7)$$

$${\dot{Q}}_{su1}=\varepsilon_{su}\cdot {\dot{m}}_{r}\cdot c_{p,su}\cdot (T_{w}-T_{su})\;(8)$$

$$\varepsilon_{su}=1-e^{-\frac{AU_{su}}{{\dot{m}}_{r}\cdot c_{p,su}}}\;(9)$$

where *Ċ _su_* is the fluid heat capacity rate, *c _p,su_* is the specific heat capacity at suction conditions, *T _su1_* is the refrigerant temperature after the suction heating up, *T _su_* is the refrigerant temperature at the compressor inlet, *T _w_* is the fictitious wall temperature, and *ε _su_* is the heat transfer effectiveness at the suction port.

An appropriate correlation of the heat transfer coefficient *AU* as a function of the refrigerant mass flow rate is necessary to minimize the error on the predicted mass flow rate ^20^. Hence, a power exponent of 0.8 is typically used according to the Reynolds analogy in a fully developed turbulent flow. In that manner, the *AU* coefficients for the suction and exhaust are calculated by Equation (10).


$$AU_{(su,ex),cp}=AU_{(su,ex),cp,n}{\left[\frac{{\dot{m}}_{r}}{{\dot{m}}_{r,cp,n}}\right]}^{0.8}\;(10)$$

where *AU _su,cp,n_* and *AU _ex,cp,n_* are the nominal heat transfer coefficients at suction and discharge respectively related to the nominal mass flow rate *ṁ _r,cp,n_*.

In order to calculate heat transfer between the fluid and the casing, a fictitious isothermal wall is introduced, assumed to be constant on the entire surface of the compressor. The ambient losses are then determined by Equation (11) considering an overall heat transfer coefficient, *AU _amb_*, between the fictitious wall and compressor surroundings (of ambient temperature, *T _amb_*).


$${\dot{Q}}_{amb}=AU_{amb}\cdot (T_{w}-T_{amb})\;(11)$$

In addition, mechanical losses are transferred to the fictitious wall, and they are calculated with the use of an energy balance expressed by Equation (12).


$${\dot{W}}_{cp,loss}-{\dot{Q}}_{su12}+{\dot{Q}}_{ex12}-{\dot{Q}}_{amb}=0\;(12)$$

All the *AU* values of suction, discharge and ambient are parameters of the model and they are fine-tuned, as described later.

Pressure drop

After isobaric heating up, the isenthalpic ( *dh=0*) pressure loss simulates the pressure drop effect in the suction area of the compressor. The numerical model of this process is based on a methodology proposed by Tello-Oquendo *et al.* ^24^. The expression used is given by Equation (13) and is similar to the standard Darcy-Weisbach equation with the difference that the friction factor is not dimensionless, but rather it includes all the geometric characteristics that affect the pressure drop.


$$\Delta P_{suc}=\xi \cdot \rho \cdot \frac{u^{2}}{2}=\xi \cdot \rho \cdot \frac{{\left(n_{vol}{\dot{V}}_{s}\right)}^{2}}{2\cdot A_{su}^{2}}=K\cdot \frac{\rho }{2}\cdot {\left(n_{v}{\dot{V}}_{s}\right)}^{2}\;(13)$$

where *u* is the inlet velocity of the refrigerant, *A _su_* is the effective area at the suction port, *n _vol_* is the volumetric efficiency, and *K* is the friction factor parameter that takes into account all the geometric features and is a model parameter to be calculated.

Concerning the pressure loss at the discharge port, it is modeled as an isentropic flow ( *ds=0*) through a converging nozzle followed by an isobaric diffuser for total enthalpy recovery. The mass flow rate that exits the nozzle is related to the pressure drop and it is calculated by combining the continuity equation with the throttled density and enthalpy ( Equation (14)).


$${\dot{m}}_{r,cp}=A_{ex}\cdot \rho_{thr,ex}\cdot \sqrt{2\cdot (h_{ex2}-h_{thr,ex})}\;(14)$$

where *A _ex_* is the nozzle throat area at the discharge port, *h _ex2_* is the total specific enthalpy after the discharge pressure drop and *ρ _thr,ex_* ( Equation (15)) and *h _thr,ex_* ( Equation (16)) are the density and specific enthalpy at the throat respectively.


$$\rho_{thr,ex}=\rho (P_{ex},s_{ex2})\;(15)$$

$$h_{thr,ex}=h(P_{ex},s_{ex2})\;(16)$$

Internal leakages

There are two basic leakage paths in scroll compressors, the radial and the tangential ones. Radial leakages occur at the clearance between the scrolls and the top or bottom plate. Tangential leakages are induced by the clearance between the sidewalls of the scrolls. In order to model efficiently these leakage paths, an isentropic quasi-1-dimensional flow through a convergent nozzle is assumed. According to this, the continuity equation gives Equation (17).


$$\frac{dA}{A}=(M^{2}-1)\frac{du}{u}\;(17)$$

where *A* is the cross-section area of the nozzle, *M* is the Mach number, and *u* is the velocity of the fluid. If Mach number equals 1, then $\frac{dA}{A}=0$ which minimizes the duct area. In a convergent nozzle, *M=1* is possible only at the section of the smallest area, which is called the nozzle throat.

With known upstream pressure and temperature values, choking phenomena may occur imposing a fixed mass flow rate in the throat, when the downstream pressure is reduced to a critical level. It is evident that the maximum mass flux occurs at the location where *M=1*, with the pressure being equal to the critical value, *P _crit_* ( Equation (18)).


$$P_{crit}=P_{0}\cdot {\left(\frac{2}{\gamma +1}\right)}^{\frac{\gamma }{\gamma +1}}\;(18)$$

where *P*_0_ is the total upstream pressure, and *γ* is the isentropic exponent. 

Therefore, the leakage mass flow rate is affected by the thermodynamic conditions at the inlet and outlet of this fictitious isentropic nozzle, reforming the critical pressure with Equation (19). 


$$P_{thr,leak}=max[P_{su3},P_{crit}]=max\;\left[P_{su3},P_{ex1}\cdot {\left(\frac{2}{\gamma +1}\right)}^{\frac{\gamma }{\gamma +1}}\right]\;(19)$$

By identifying the throat pressure, the local fluid velocity is calculated via the definition of stagnation enthalpy, given by Equation (20) and Equation (21).


$$h_{ex1}=h_{\left(P_{thr,leak},s_{ex1}\right)}+\frac{1}{2}\rho_{\left(P_{thr,leak},s_{ex1}\right)}u_{thr,leak}^{2}\;(20)$$

$$u_{thr,leak}=\sqrt{2(h_{ex1}-h_{\left(P_{thr,leak},s_{ex1}\right)})}\;(21)$$

The leakage mass flow rate, which relates density, velocity, and effective area is then calculated by Equation (22).


$${\dot{m}}_{r,leak}=\rho \cdot u\cdot A=A_{leak}\cdot \rho_{thr,s_{ex1}}\cdot \sqrt{2\left(h_{ex1}-h_{\left(P_{thr,leak},s_{ex1}\right)}\right)}\;(22)$$

where *A _leak_* is the effective cross-sectional area of the nozzle throat, which is a parameter of the model to be identified.

Finally, after determining the leakage mass flow rate, *ṁ _r,leak_*, the thermodynamic state of the refrigerant at the compressor inlet is calculated with Equation (3) and the energy conservation equation for mixing ( Equation (23)).


$${\dot{m}}_{r}\cdot h_{su2}+{\dot{m}}_{r,leak}\cdot h_{ex1}={\dot{m}}_{r,cp}\cdot h_{su3}\;(23)$$

***Performance indicator.*** An expression for the isentropic efficiency is used to evaluate the performance of the scroll compressor at different operating conditions. Isentropic efficiency gives the ratio between the ideal and actual power consumption of the compressor. This indicator is provided by Equation (24).


$$n_{cp}=\frac{{\dot{m}}_{r,cp}\;(h_{ex,is,cp)}-h_{su,cp}}{{\dot{W}}_{cp}}\;(24)$$

where *h _ex,is,cp_* is the outlet enthalpy once the refrigerant is compressed isentropically, and *h _su,cp_* is the enthalpy at the beginning of compression.

***Model parameters.*** The model predicts the compressor discharge temperature, the refrigerant flow rate, the ambient losses, and the compressor electrical power by using as inputs the condensing and evaporating temperatures, ambient temperature, and compressor speed and displacement. All the equations described above, formulate the compressor model and introduce a set of parameters that has to be identified to match the numerical results with the data obtained from experiments or from the compressor manufacturer. In this study, the total number of parameters is 12 and they are presented in Table 2.

**Table 2.** Parameters of the semi-empirical model and relevant compressor sub-process.

Parameter	Units	Sub-process
*AU _su,cp,n_*	W/K	Heating-up during supply
*AU _ex,cp,n_*	W/K	Heat loss during the exhaust
*AU _amb_*	W/K	Heat losses to the ambient
*A _leak_*	m ^2^	The effective area of leakages
*Ẇ _cp,loss,0_*	W	Constant term of the electrical losses
*α _cp_*	-	Coefficient of the variable term of the electrical losses
*d _ex_*	m	Exhaust diameter for discharge pressure losses
*corr*	-	Motor slip coefficient
*r _v,in_*	-	Build-in volume ratio of the compression process
*ṁ _r,cp,n_*	kg/s	Nominal mass flow of the refrigerant for heat transfer calculations
*V _s, cp_*	m ^3^	The swept volume of the compressor
*K*	1/m ^4^	Friction factor for suction pressure drop

From the parameters of Table 2, the diameter *d _ex_* can be found in the manufacturer’s technical drawings, and *corr* is obtained from the literature ^22, 25^. The reference mass flow rate is calculated by multiplying the compressor swept volume by the density defined at thermodynamic state with saturated vapor at 0 °C. The remaining 9 parameters need to be calibrated from available data preferably over a large range of evaporation and condensation temperatures. This procedure is different for each method examined here, although there are some common aspects, as described next.

### Method 1 – complete set of parameters

For calibrating all model parameters, an optimization method has been followed with genetic algorithms for minimizing the relative difference of discharge temperature, electricity consumption, and refrigerant mass flow rate, under the Engineering Equation Solver (EES) environment ^26^. For that purpose, a function *Θ* is defined by Equation (25), which needs to be minimized. No weighting factor has been used for the three components of this function.


$$\Theta =\sqrt{\frac{1}{Z}\sum_{l=1}^{Z}\left[{\left(\frac{T_{calc,i}-T_{data,i}}{T_{data,i}}\right)}^{2}+{\left(\frac{{\dot{m}}_{calc,i}-{\dot{m}}_{data,i}}{{\dot{m}}_{data,i}}\right)}^{2}+{\left(\frac{P_{calc,i}-P_{data,i}}{P_{data,i}}\right)}^{2}\right]}\;(25)$$

In Equation (25), *Z* is the number of *i* data sets considered for the optimization, and the subscripts *calc* and *data* correspond to the calculated values and the available data respectively.

**Figure 3.**
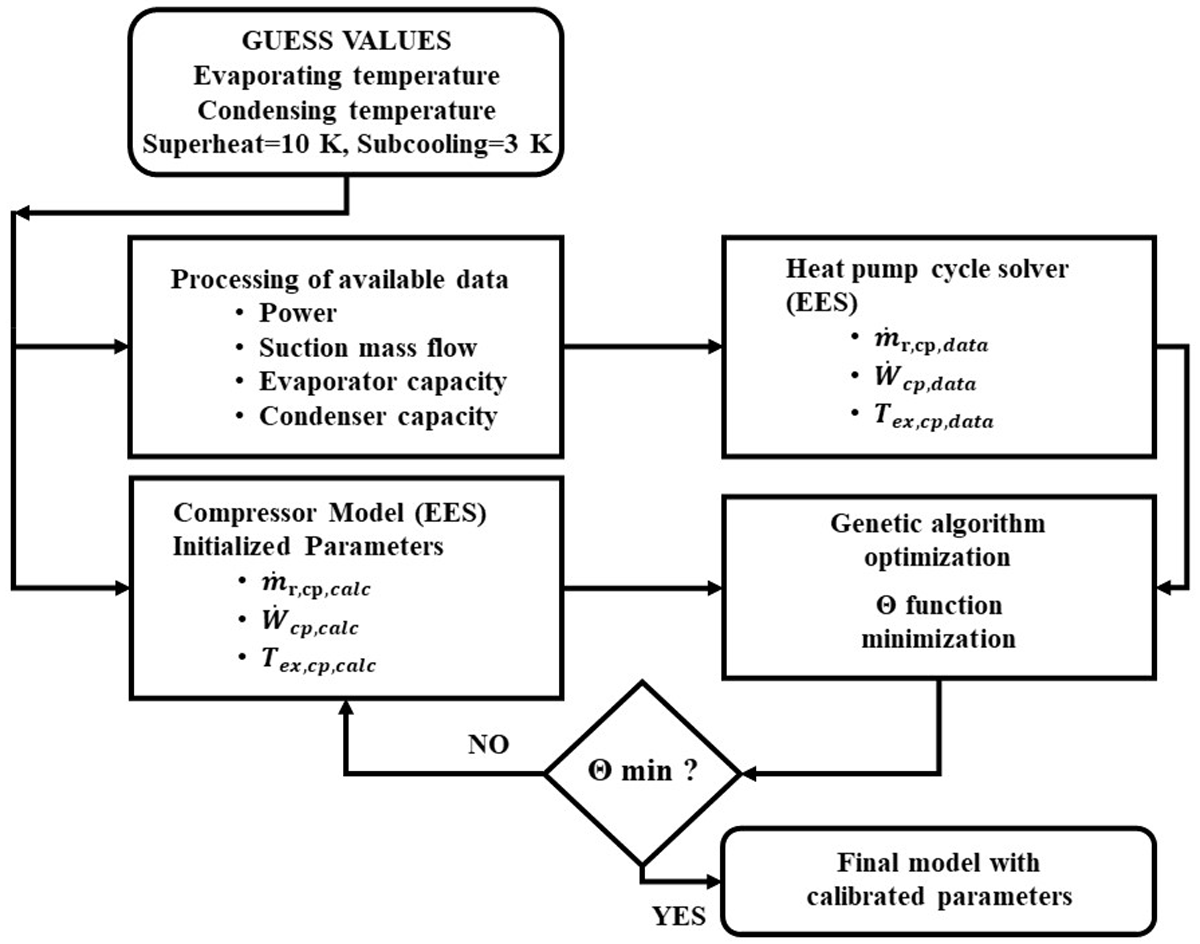
Model parameters calibration flow chart.

The computational methodology for parameters identification demands a standard set of variables including evaporating and condensing temperatures, suction superheat (set to 10 K), rotational compressor speed (always kept constant and equal to 3000 rpm), and condenser subcooling (set to 3 K), in order to form the compressor operation range. With these inputs, the available data provide all the thermodynamic and performance indicators that make it possible to calculate the inlet and outlet states of the refrigerant. In consequence, by inserting these data in the compressor model it is possible to identify all the parameters of method 1 by minimizing the function of Equation (25). By doing so, the calibrated model is compatible with each combination of compressor and refrigerant. The flow chart of this process is presented in Figure 3.

### Method 2 – reduced set of parameters

The model described previously simulates the performance of a certain compressor and refrigerant after determining the set of parameters. This methodology can be simplified when changing the refrigerant, by keeping constant most of the parameters for a specific compressor that are not refrigerant-specific, performing the optimization process only for a subset of the parameters. The compressor-specific parameters, such as build-in volume ratio, leakage area, swept volume and exhaust diameter are then kept constant. Τhe same applies to the model parameters related to the power indicators of the compressor motor, such as *Ẇ _cp,loss,0_* and *α _cp_*. Therefore, the examined set of parameters is reduced to four that includes the following: *AU _amb_*, *AU _ex,cp,n_*, *AU _su,cp,_*_n,_ and the friction factor *K*.

By using the same *N* data sets, the calibration process is performed initially with the reference refrigerant for the total number of parameters and for a different refrigerant it is performed only for the reduced set of this second method. This leads to the reduction of the necessary computational time of the overall procedure by approximately 60% compared with method 1. However, method 2 requires the use of a reference case, in order to extract the parameters that are then kept constant.

### Method 3 – refrigerant specific parameters from correlations

The third method adapts the fluid altering process without the need of any optimization method, simplifying and expediting the calculations with a more generalized methodology for any working fluid, by adjusting the refrigerant-related parameters. This procedure was inspired by Byrne *et al.* ^16^ and Duprez *et al.* ^17^ on scroll compressors and Muye *et al.* ^19^ and Guiffrida ^27^ on scroll expanders respectively.

In order to adapt the fluid altering process, the heat transfer coefficients ( *AU*) of suction and discharge need to be recalculated ^16^. On the other hand, the ambient heat losses are assumed to have no dependency on the refrigerant because represent the heat transfer from the casing to the surrounding environment. Moreover, the overall leakage area does not vary with the fluid switch. This also applies to the build-in volume ratio and the swept volume, which are intrinsic characteristics of the examined machine. As far as friction losses are concerned, both working fluid and operating conditions (pressure ratio and rotational speed) are associated with the calculations. The density of the reference refrigerant and its replacement as well as their ratio has an effect on the friction losses ^19^. Considering this, the model takes this into account, by including the refrigerant density in Equation (14), and thus the friction factor is assumed to have a constant value for any refrigerant change. The equations that are included in the model of this process are given by Equation (26)– Equation (29) ^27^, resulting to Equation (30).


$$U=\frac{Nu\cdot \lambda }{L}\;(26)$$

$$Nu=0.023\cdot Re^{0.8}\cdot Pr^{m}\;(27)$$

$$Re=\frac{\rho \cdot u\cdot D}{\mu }\;(28)$$

$$Pr=\frac{c_{p}\cdot \mu }{\lambda }\;(29)$$

$$AU_{fluid}=AU_{ref,fluid}\cdot {\left(\frac{\rho_{fluid}}{\rho_{ref,fluid}}\right)}^{0.8}\cdot {\left(\frac{\mu_{ref,fluid}}{\mu_{fluid}}\right)}^{0.8-m}\cdot {\left(\frac{c_{p_{fluid}}}{c_{p_{ref,fluid}}}\right)}^{m}\cdot {\left(\frac{\lambda_{fluid}}{\lambda_{ref,fluid}}\right)}^{1-m}\;(30)$$

where *U* is the overall heat transfer coefficient, *Re* and *Pr* are the Reynolds and Prandtl number of the flow, *λ* is the thermal conductivity, and *μ* is the dynamic viscosity. The characteristic length *L* and the hydraulic diameter *D* in Equation (28) and Equation (28) respectively are constant for any fluid because they depend only on the scroll geometry. Furthermore, the velocity *u* is assumed to be constant, because it is related to the passage geometry and the swept volume of the compressor. The Nusselt number *Nu*, is calculated by Dittus and Boelter heat transfer correlation for turbulent flow in smooth pipes with the exponent *m* equal to 0.3 if *T _w_<T _fluid_* and to 0.4 if *T _w_>T _fluid_* ^19^.

Applying this method 3 to the semi-empirical model, it is possible to determine the model performance for any working fluid avoiding the fluid-by-fluid optimization process that is required in the previous two methods. Instead of that, a certain refrigerant is set as a reference, and for any other operation with a different refrigerant the parameters *AU _su_* and *AU _ex_* are only calculated. This process is very fast since the optimization method is applied only once, further shortening the computational time to a few seconds for the model adaptation to different refrigerants. By doing so, the calculation time is reduced from 90 minutes with method 1 and 35 minutes with method 2 to less than 0.1 minutes with method 3. All the calculations were performed with an Intel Core i-3 dual-core 2.4 GHz processor.

### Examined cases for methods verification

In order to verify the compressor model accuracy and the three methods, a comparison campaign is carried out with available data of two scroll compressors with three refrigerants for each machine, based on data available from the manufacturer. The verification focuses on recent compressor series that are suitable for both HFC and HFO refrigerants. The main specifications of the two selected compressors are shown in Τable 3, according to the manufacturer.

**Table 3.** Main specifications of the two scroll according to manufacturer data.

Compressor	Motor Nominal voltage/phase	Displacement at 50 Hz (m ^3^/h)	Discharge fitting (mm)	Available refrigerants
**YH06K1E-TFMN**	400 V / 3~	8	12.7	R134yf, R454C, R134a, R407C, R513A
**ZR36KRE-TFD**		8.61		R134a, R450A, R513A, R407C

For each compressor and refrigerant, a standard set of variables including evaporating and condensing temperatures, suction superheat and condenser subcooling is assumed, in order to form the compressor operation range. By doing so, the first method is initially applied, calculating the semi-empirical model parameters *via* optimization for each combination of compressor and refrigerant. The refrigerants taken into examination, are chosen from the available ones and are given in Table 4.

**Table 4.** GWP and ASHRAE class of the examined refrigerants and combinations of compressors and refrigerants.

Refrigerant	Classification	GWP	ASHRAE class	Compressor examined
**R134a**	HFC	1430	A1	YH06K1E, ZR36KRE
**R1234yf**	HFO	4	A2L	YH06K1E
**R407C**	HFC blend	1774	A1	YH06K1E, ZR36KRE
**R450A**	HFO blend	650	A1	ZR36KRE

Combining the two compressor models with the selected refrigerants, six cases are established, with the last column of Table 4 indicating which compressor is examined with the selected refrigerants.

### Summary of methods based on the refrigerant-compressor pairs

By using the refrigerant-compressor combinations of Table 4, the defined reference refrigerant for methods 2 and 3 is the R134a. Therefore, the cases combination of R134a with each compressor results to the same for every method, since the standard optimization methodology is used to identify the reference set of parameters. Based on this, the method comparison is reasonable only for the cases with refrigerants other than R134a, leading to two cases for each compressor to provide a comparison between all three methods.

For the sake of brevity, the considered methodologies are summarized in Table 5, as method 1 with the complete set of parameters, method 2 for the reduced set, and method 3 for the generalization method. In the next paragraphs, the reference for these methods will be done by the following shortcuts.

**Table 5.** Highlights of the considered methodologies.

Method	Highlights
**1**	Semi-empirical model with the complete set of parameters and optimization fluid–by–fluid for every compressor and refrigerant for parameters identification.
**2**	Semi-empirical model with a reduced set of parameters, once a reference case is identified. Performing optimization only for *AU _amb_*, *AU _su_*, *AU _ex_*, and *K* with the other parameters constant for the fluid change.
**3**	Generalization model with constant parameters, once a reference case is identified, and recalculating *AU _su_*, *AU _ex_* for refrigerant change.

## Results and discussion

In order to verify the performance of the established semi-empirical model for scroll compressors, the YH compressor is examined (reference compressor), initially focusing on method 1. After that, the results of the other two methods are presented and compared, once the refrigerant is changed. Finally, the same procedure has been followed for the ZR compressor, with the aim to expand the outcomes and conclusions related to the applicability of the three methods.

### Semi-empirical model accuracy with the reference refrigerant – method 1

By using the available data of the manufacturer, the compressor performance is examined in a wide operating range. In total, 20 testing points have been used for that case, covering evaporation temperatures from −20 to 15°C and condensation temperatures from 20 to 60°C. This range corresponds to a wide variety of applications, spanning from refrigeration to heat pumps. The corresponding pressure ratios have a sufficient range from 2 to 7.65, enclosing a wide area of the working envelope.

Initially, the reference mass flow rate is identified as well as the parameters *corr* and *d _ex_*, according to the proposed methodology. Their values are 0.03208 kg/s, 0.04, and 12.7 mm respectively. The numerical model is then applied for fine-tuning all the parameters through the optimization process previously described.

**Figure 4.**
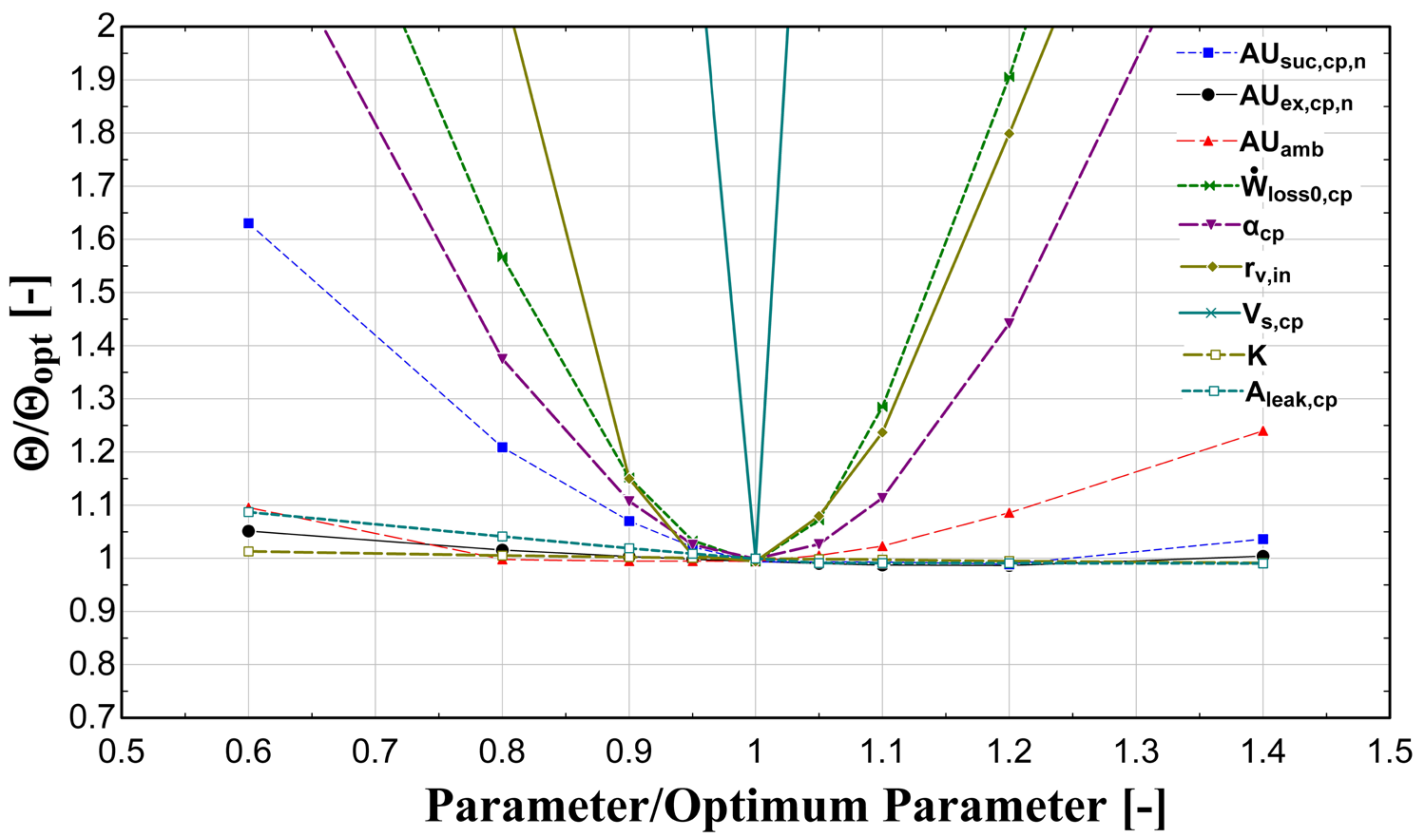
Sensitivity analysis of calibrated model parameters.

The optimization method is repeated with different guess values and a sensitivity analysis is implemented by varying the identified parameters between ±40%, in order to find the global minimum of the *Θ* function. The results of this process are presented in Figure 4.

This analysis shows that the *AUs* have a weak effect on the function minimization. This is a critical outcome because in case these are calculated by the fluid change methodology (method 3), a small variation compared with the global optimized one is expected. On the other hand, the model seems to have a high sensitivity on the compressor swept volume, built-in volume ratio, and electrical-related parameters. It should be highlighted here that similar trends and effects are obtained from other relevant studies in the literature ^21, 25, 28^. This process resulted to the calculated parameters for the reference case with R134a (method 1), which are given in Table 6.

**Table 6.** Calculated parameters of method 1 with the YH compressor and R134a.

Parameter	Value	Units
*AU _amb,cp_*	2.48	W/K
*AU _ex,cp,n_*	12.23	W/K
*AU _su,cp,n_*	20.62	W/K
*A _leak, cp_*	5.21E-08	m ^2^
*Ẇ _loss0,cp_*	175.10	W
*α _cp_*	0.17	-
*V _s,cp_*	46.54	cm ^3^
*r _v,in_*	3.40	-
*K*	2.71E+07	1/m ^4^

The compressor displacement of 8 m ^3^/h corresponds to a swept volume of 44.44 cm ^3^ for a rotational speed of 3000 rpm. However, the calculated swept volume is 4.7% higher than that. Such small variation could be expected and is acceptable, since the calculated swept volume is something fictitious taking into consideration characteristics that the sub-processes of the semi-empirical model do not include, such as phenomena related to the oil flow or over-compression effect at the suction port ^8^. Another reason could be related to the compression of the fluid during the suction process, due to the decrease of the volume in the pockets. This overestimation is very well aligned with the findings of several other studies, such as of Dardenne *et al.* ^21^ who calculated a higher swept volume by 1.5%, Winandy *et al.* ^20^ by 6%, and Cuevas *et al.* ^22^ by 4.5%.

**Figure 5.**
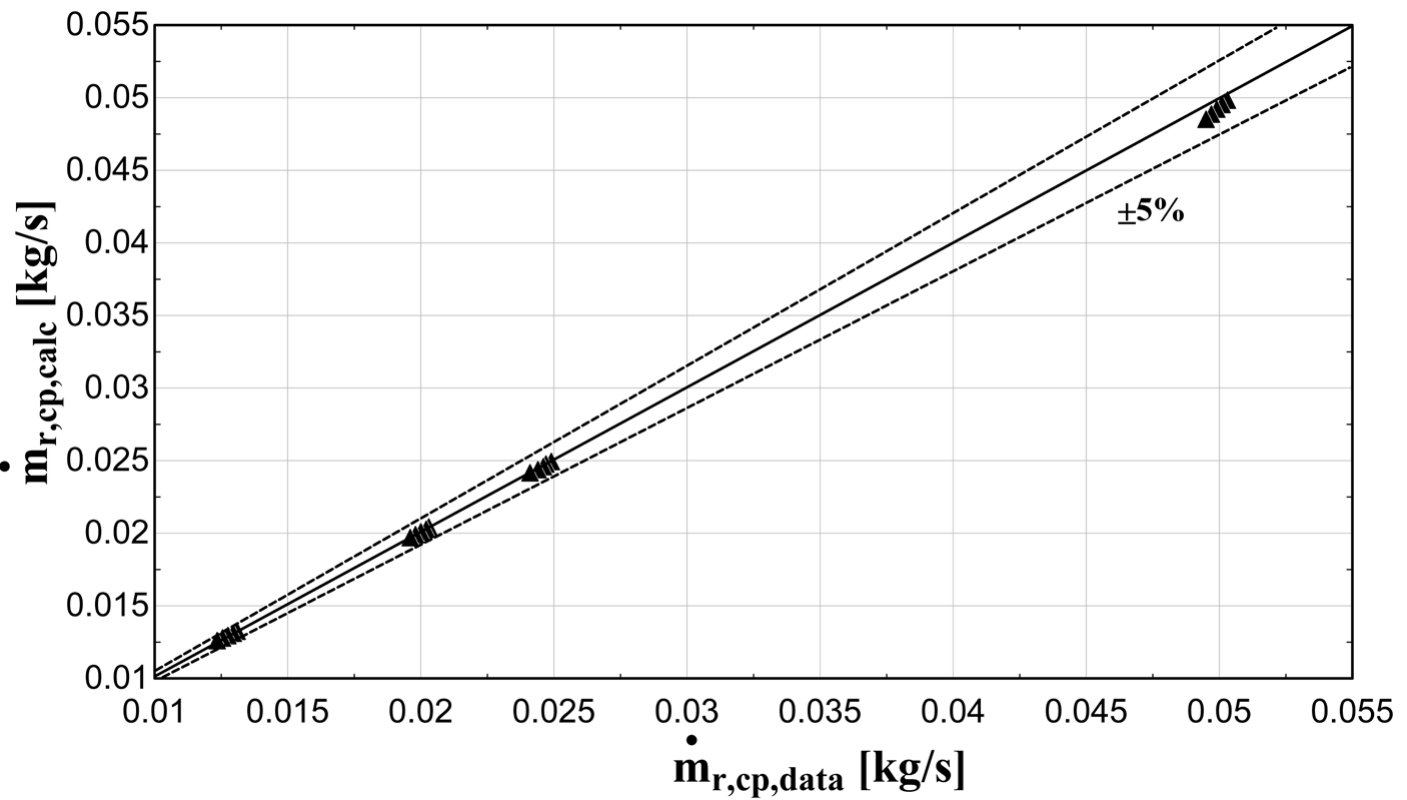
Comparison between the calculated suction mass flow rate and the available one.

The comparison of the calculated suction mass flow, the electric power, the refrigerant discharge temperature, and the isentropic efficiency of method 1 with the available data is presented in Figure 5– Figure 8 respectively, also indicating the curves with a relative variation of ±5% (for discharge temperature the relative variation curves are for ±3 K).

**Figure 6.**
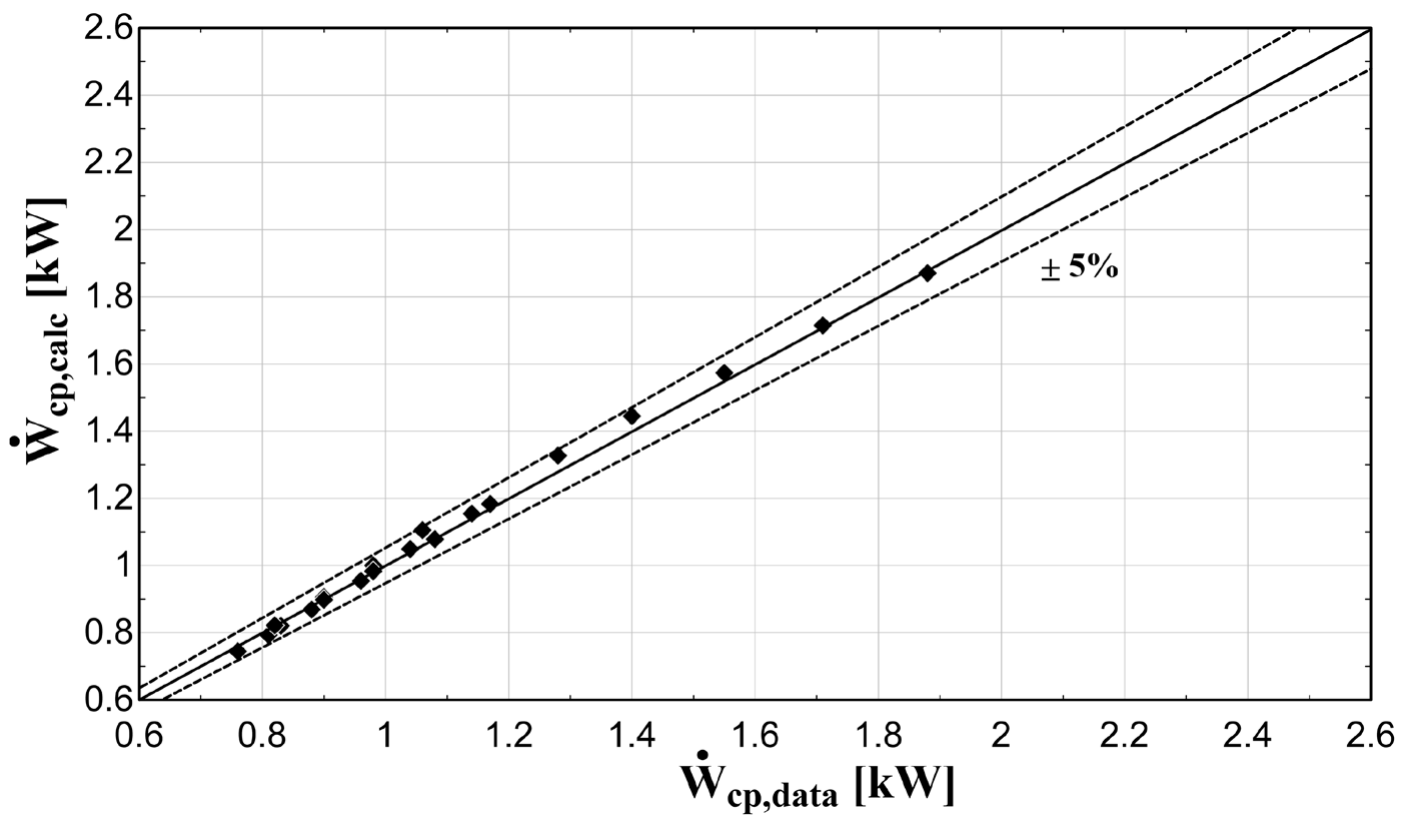
Comparison between the calculated electric power and the available one.

**Figure 7.**
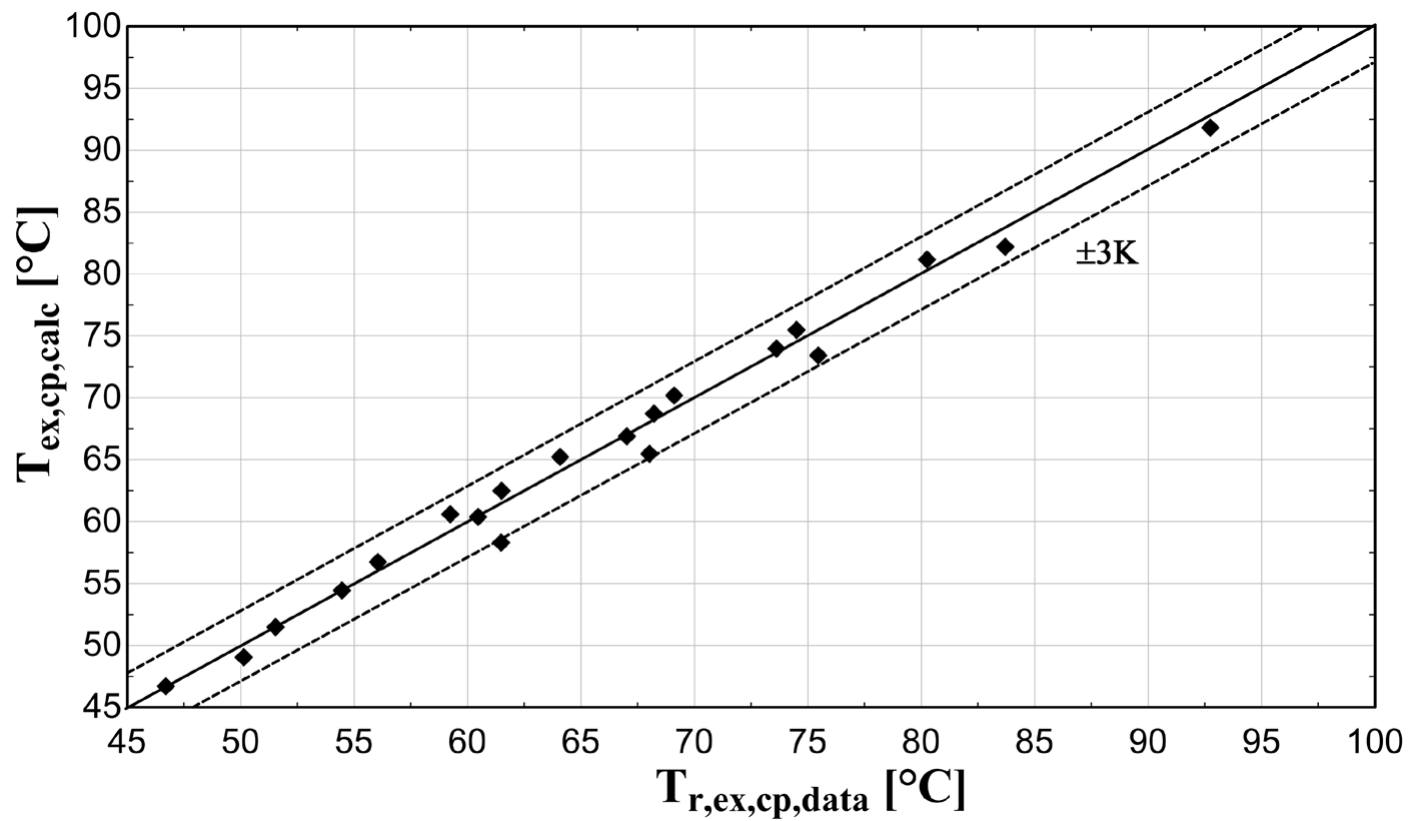
Comparison between the calculated discharge temperature and the available one.

**Figure 8.**
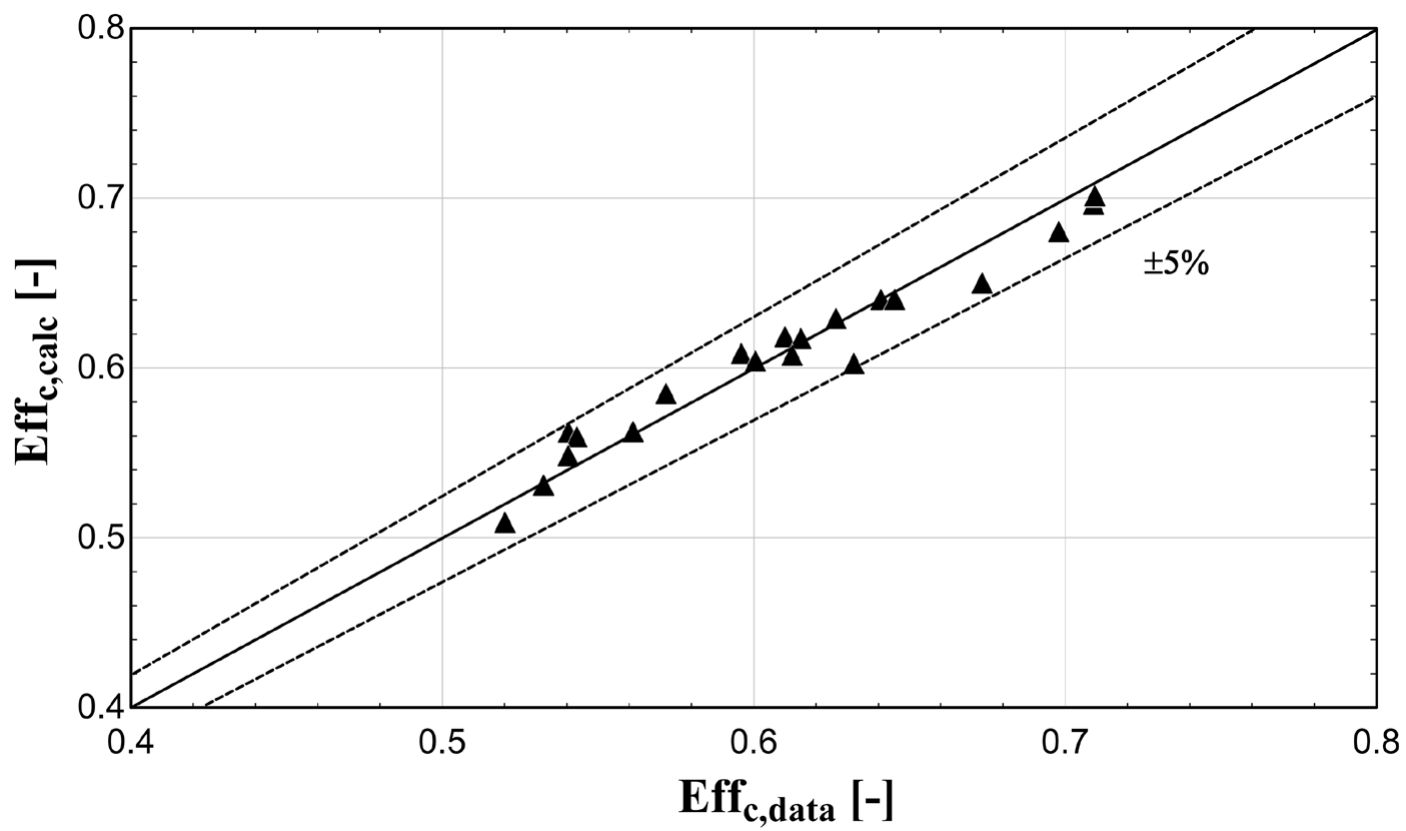
Comparison between the calculated isentropic efficiency and the available one.

The model always predicts the suction mass flow rate, the power, and the isentropic efficiency within a 5% margin from the available data. At low/moderate refrigerant mass flow rates, the model accuracy is even better, as depicted in Figure 5 This brings a better prediction accuracy to the power consumption, which is highlighted in Figure 6, although the differences are small. On the other hand, the discharge temperature shows about the same high accuracy all over the range of the operating conditions, which is within the ±3 K range. Finally, the isentropic efficiency depends from all previous variables and a higher deviation of the calculated values from the available ones is observed. But still, the prediction accuracy is within 5% from the available data.

The minimum and maximum deviation of the calculated values of all four key variables compared with the available data is given in Table 7.

**Table 7.** Minimum and maximum deviation of the calculated key variables of method 1 compared with the available data.

Temperature deviation (K)		Mass flow rate deviation (%)		Electric power deviation (%)		Isentropic efficiency deviation (%)	
Min	Max	Min	Max	Min	Max	Min	Max
−3.18	1.35	−1.99	2.06	−2.08	4.27	−4.67	4.02

### Methods comparison with YH compressor

After the semi-empirical model fine-tuning and verification with the reference compressor (YH) and refrigerant (R134a), the comparison between the considered methods takes place. With the same compressor, the parameters are identified using each methodology respectively, concerning the operation with R407C and R1234yf. The results with R134a are the same for every case, since it is the reference refrigerant. Table 8 provides the parameters of the semi-empirical model, calculated by applying all three methods for the refrigerant change. The second column is for R134a (reference) and is the basis for the parameters identification of methods 2 and 3.

**Table 8.** Values of the parameters for YH compressor and the three refrigerants with all methods.

Parameters	Refrigerants						
	R134a	R407C			R1234yf		
	Rerefence	Method 1	Method 2	Method 3	Method 1	Method 2	Method 3
**AU _amb,cp_**	2.48	2.14	3.57	2.48	3.44	3.44	2.48
**AU _ex,cp,n_**	12.24	10.04	8.52	15.46	24.27	14.27	14.94
**AU _su,cp,n_**	20.62	22.43	30.32	26.37	22.11	32.11	25.21
**A _leak,cp_**	5.21E-08	5.21E-08			5.21E-08		
**Ẇ _loss0,cp_**	175.10	105.90	175.10		175.00	175.10	
**α _cp_**	0.17	0.24	0.17		0.27	0.17	
**V _s,cp_**	46.54	46.75	46.54		47.00	46.54	
**r _v,in_**	3.40	3.42	3.40		3.44	3.40	
** $\dot{m}$ _r,cp,n_**	0.032	0.044			0.039		
**K**	2.71E+07	1.55E+08	1.04E+08	2.71E+07	2.81E+07	2.81E+07	2.71E+07

**Figure 9.**
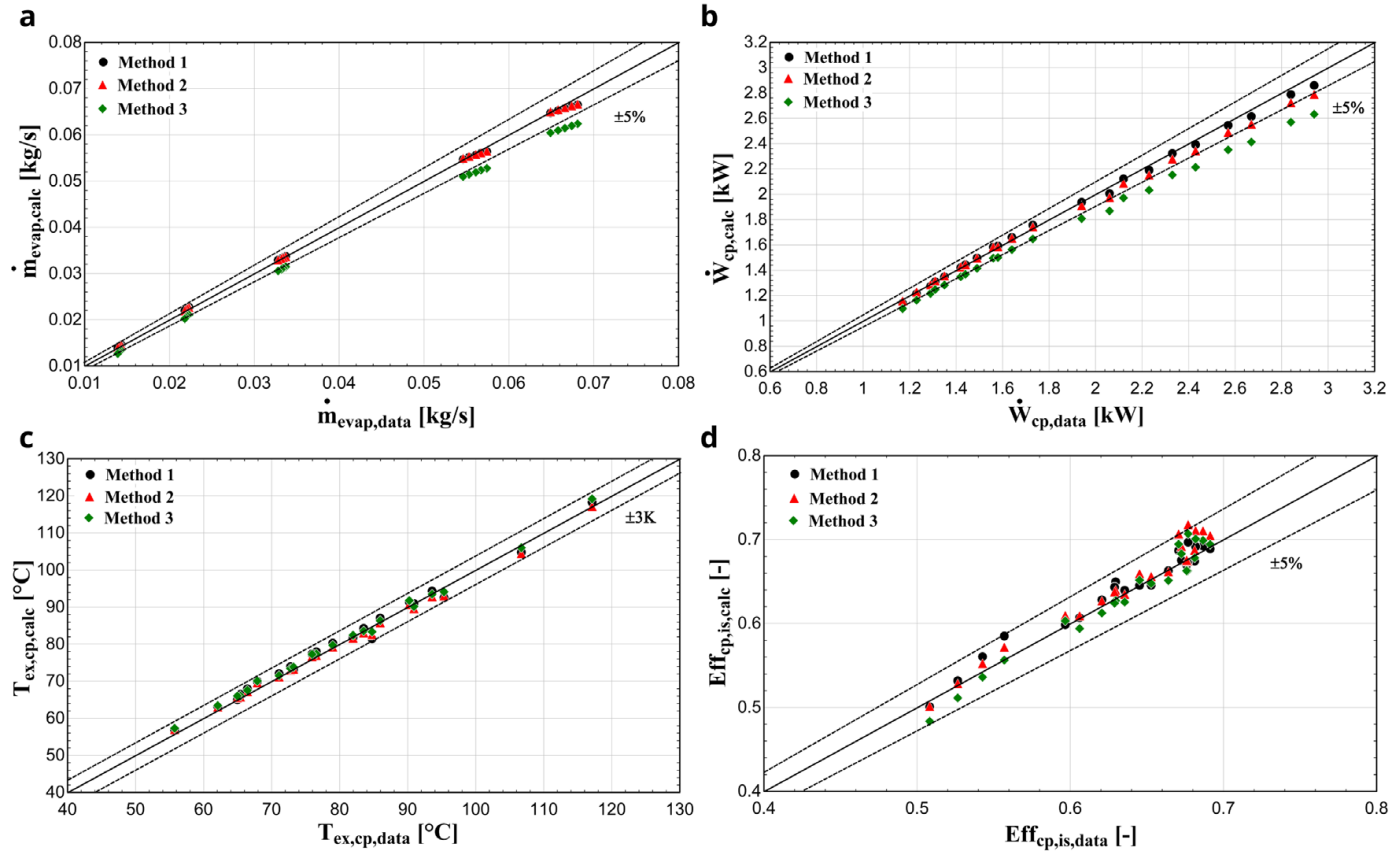
( **a**) Mass flow rate, ( **b**) electric power, ( **c**) discharge temperature, and ( **d**) isentropic efficiency comparison with the YH compressor and R407C for all three methods.

***Results with R407C.*** The comparison of the calculated key results with the available data for the 22 operating conditions with the R407C and the YH compressor for all three methods is shown in Figure 9.

The discharge temperature is sufficiently calculated with all three methods, keeping the deviation within ±3 K for all conditions. This is the outcome of the low sensitivity of the function to be minimized from the *AUs*, as shown in Figure 4, because these parameters regulate the heat gain and loss during supply and discharge respectively. Especially the latter has a major impact on the discharge temperature, without affecting any other sub-process. Also, the isentropic efficiency calculated by the semi-empirical model shows a good compliance with the data provided by manufacturer, maintaining the deviation within the ±5 % range for all methods.

However, method 3 seems to be less accurate for predicting the mass flow rate and the electric power, underestimating these variables for all conditions and in many cases exceeding the 5% limit and reaching even 10%. The refrigerant’s mass flow rate is primarily regulated by the swept volume and secondarily by the *AU _su_* and friction factor as they affect the fluid density at the compressor suction port. These variables are identified based on the reference refrigerant with method 3, resulting to a less accurate prediction compared with method 1. The same also applies to the electric power as the dominant parameters are *α _cp_*, and *Ẇ _loss,0_*, which are kept constant when the refrigerant changes. Furthermore, electric power accuracy is related to the mass flow rate, leading to a lower accuracy, when the latter is not predicted with an adequate accuracy. The parameters such as V _s,cp_, Κ, α _cp_, and Ẇ _loss,0_ do not vary for each fluid change, not being refrigerant specific parameters, but actually, the semi-empirical model accuracy is enhanced when these parameters are recalculated for every refrigerant calibration. This could be related to the assumptions of the model as well as the systematic and random errors of the data set used for the calibration.

Finally, method 2 provides more accurate results than method 3 in general, giving similar performance regarding the mass flow rate since the *AU _su_* and the friction factor are refrigerant-specific parameters and are identified for the certain refrigerant. Again, a small underestimation of the electric power is observed, reaching 5% for low evaporation temperatures (high electric power), because *α _cp_*, and *Ẇ _loss,0_* are also kept constant.

**Figure 10.**
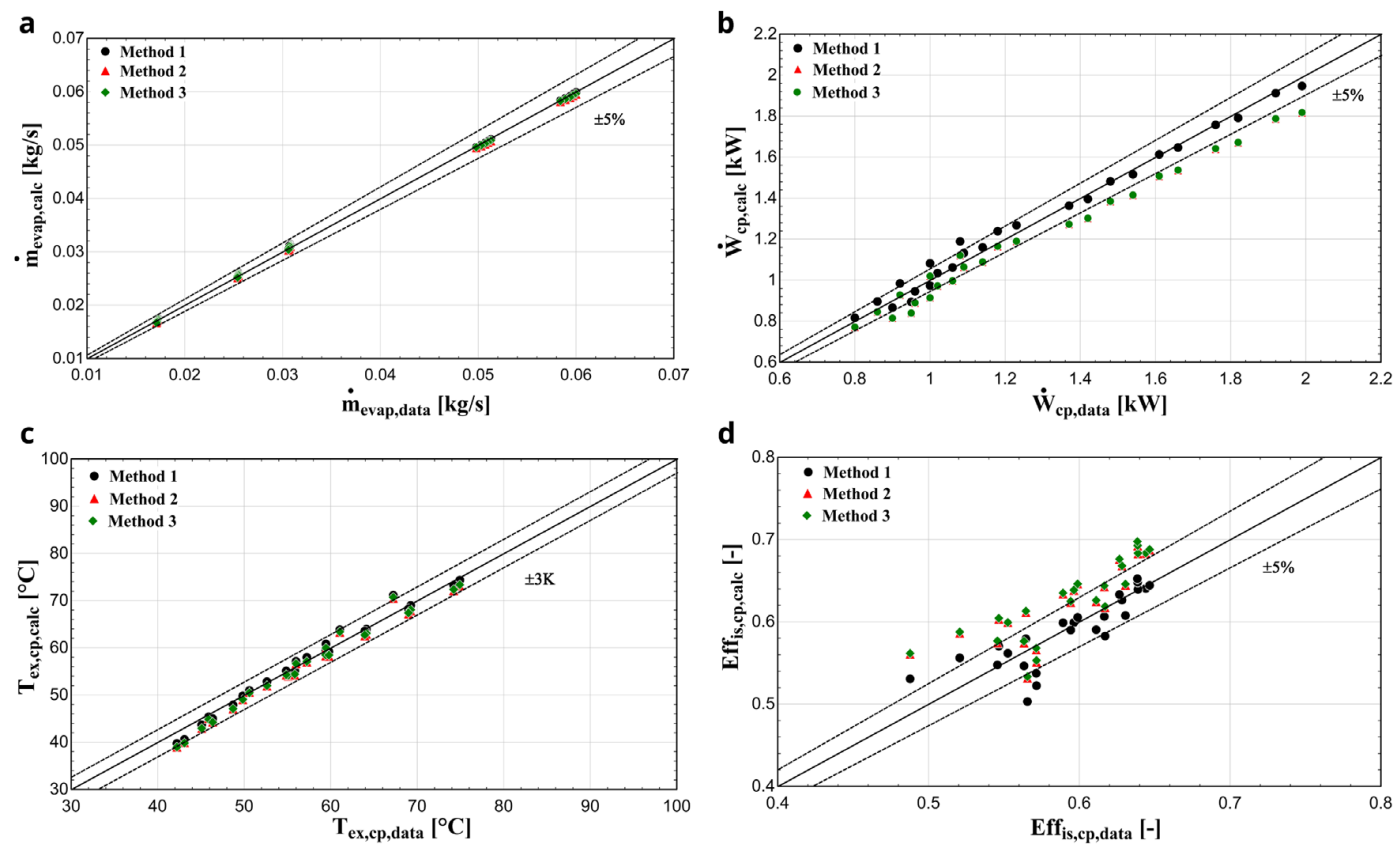
(a) Mass flow rate, (b) electric power, (c) discharge temperature and (d) isentropic efficiency comparison with YH compressor and R1234yf for all three methods.

***Results with R1234yf.*** The three considered methodologies are also compared when R1234yf refrigerant is used. In that case, the comparison diagrams of the mass flow rate, electric power, discharge temperature and isentropic efficiency are depicted in Figure 10.

Once again, method 1 provides the highest accuracy with its results being almost identical to the available data. The mass flow rate is very well predicted for all three methods, which was not the case with R407C. This can be explained, because R1234yf has very similar thermophysical properties to the reference refrigerant R134a, such as almost identical saturation pressures, molar mass and critical temperature ^29^. On the other hand, the saturation pressures of R407C for various temperatures are higher compared with the ones of R134a ^30^. The calculated electric power of methods 2 and 3 is underestimated by up to about 10%, while the results of method 1 match the available data, especially for reduced evaporation temperatures. Finally, method 1 predicts the isentropic efficiency with much better accuracy with almost all conditions included within the ±5 % range compared with methods 2 and 3, in which this efficiency is overestimated by up to 10–12%.

***Overall accuracy of each method with the YH compressor.*** Comparing the model results for every refrigerant with the data from the manufacturer, it is possible to investigate the overall accuracy of each method. The absolute relative error is determined with Equation (31) and the standard deviation with Equation (32) ^22^.


$$\left|{\bar{\varepsilon }}_{rel}\right|=\frac{1}{N}\sum_{i=1}^{N}\left|\frac{\left(X_{i,data}-X_{i,calc}\right)}{X_{i,data}}\right|\;(31)$$

$$\sigma ={\left[\frac{1}{N}\sum_{i=1}^{N}{\left(\varepsilon_{i}-{\bar{\varepsilon }}_{l}\right)}^{2}\right]}^{0.5}\;(32)$$

where the *X* parameter corresponds to the discharge temperature, electric power, refrigerant mass flow rate and isentropic efficiency of the *i* condition, and the subscripts *data* and *calc* refer to the available data and the calculations respectively.

Table 9 gives the average absolute error and standard deviation of the calculated values compared with the available data for every method and refrigerant operation respectively.

**Table 9.** Error analysis of the considered methodologies with R407C and R1234yf.

		R407C			R1234yf		
		Method 1	Method 2	Method 3	Method 1	Method 2	Method 3
**Relative** **average** **error**	Discharge temperature (°C)	1.21	**0.80**	0.97	**0.98**	1.52	1.43
	Electric power (%)	**1.15**	1.85	6.68	**2.79**	5.91	5.80
	Mass flow rate (%)	1.03	**0.81**	7.19	**0.76**	0.88	0.77
	Isentropic efficiency (%)	**1.41**	1.86	1.76	**3.09**	6.35	6.48
**Standard** **deviation**	Discharge temperature (°C)	1.37	1.07	**0.95**	**1.37**	1.44	1.45
	Electric power (kW)	**0.03**	0.05	0.08	**0.04**	0.05	0.05
	Mass flow rate (kg/s)	0.0006	**0.0005**	0.0016	**0.0003**	0.0003	0.0003
	Isentropic efficiency (-)	**0.0100**	0.0123	0.0134	**0.0238**	0.0260	0.0255

The results of method 1 overall show smaller differences compared with the manufacturer data, especially with R1234yf. This is expected, since the values of all parameters are optimized for the specific refrigerant operation. Method 2 shows similar accuracy with the first method with R407C. Finally, method 3 is the least accurate, having the highest absolute average error and deviation, with its advantages the simpler calculations without any optimization process involved and without knowing any experimental or manufacturer data, and the much lower computational time required.

### Methods comparison with ZR compressor

In order to further verify the previous outcomes and ensure the wider applicability of the suggested methods, another scroll compressor is considered. The reference refrigerant is again R134a. The same three methods are applied with the refrigerant change relying on R407C and R450A.

Similar as before, the semi-empirical model parameters have been identified, and their values are presented in Table 10. Concerning the swept volume calculations, the fine-tuning process with R134a concluded to a 6.6% higher volume compared with the one provided by the manufacturer, confirming the previous remarks regarding the difference between the theoretical and the calculated swept volume.

**Table 10.** Values of the parameters for ZR compressor and the three refrigerants with all methods.

Parameters	Refrigerants						
	R134a	R407C			R450A		
	Rerefence	Method 1	Method 2	Method 3	Method 1	Method 2	Method 3
**AU _amb,cp_**	5.47	1.50	7.35	5.47	1.06	1.32	5.47
**AU _ex,cp,n_**	6.09	30.79	13.02	7.75	8.80	3.16	5.65
**AU _su,cp,n_**	37.38	34.77	57.85	47.79	26.10	29.57	34.61
**A _leak,cp_**	1.29E-08	1.29E-08			1.29E-08		
** $\dot{W}$ _loss0,cp_**	228.40	96.38	228.40		205.30	228.40	
**α _cp_**	0.15	0.15	0.15		0.18	0.15	
**V _s,cp_**	51.00	53.00	51.00		49.91	51.00	
**r _v,in_**	2.91	2.91	2.91		3.04	2.91	
** $\dot{m}$ _r,cp,n_**	0.035	0.047			0.032		
**K**	2.30E+08	2.30E+08	2.76E+08	2.30E+08	1.27E+08	1.82E+08	2.30E+08

**Figure 11.**
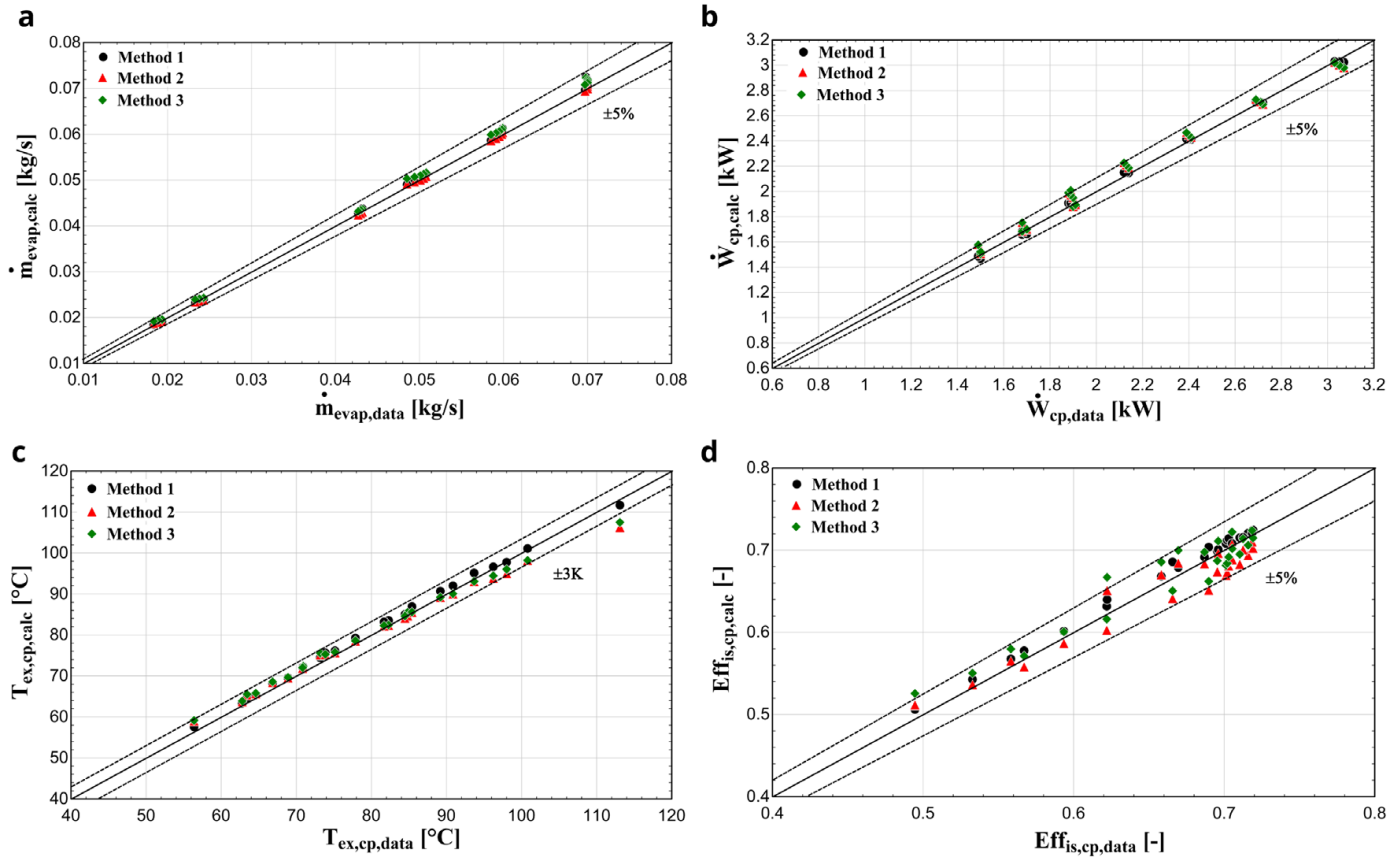
( **a**) Mass flow rate, ( **b**) electric power, ( **c**) discharge temperature, and ( **d**) isentropic efficiency comparison with ZR compressor and R407C for all three methods.

***Results with R407C.*** After the calculation of the parameters, the comparison of the results of the three methods takes place. Figure 11 depicts the results with R407C for all three methods, demonstrating similar performance with the previous compressor.

In this case, all three methodologies give similar results for the electric power and mass flow rate. Regarding the discharge temperature prediction, method 1 shows a better performance especially for higher temperatures, compared with methods 2 and 3 where a larger deviation is identified, which exceeds the ±3 K borderline in a few conditions. Due to the higher accuracy of method 1, the isentropic efficiency shows much better results compared with the other two methods. In any case, the calculated efficiency is within the ±5 % range for most of the operating conditions and methods.

**Figure 12.**
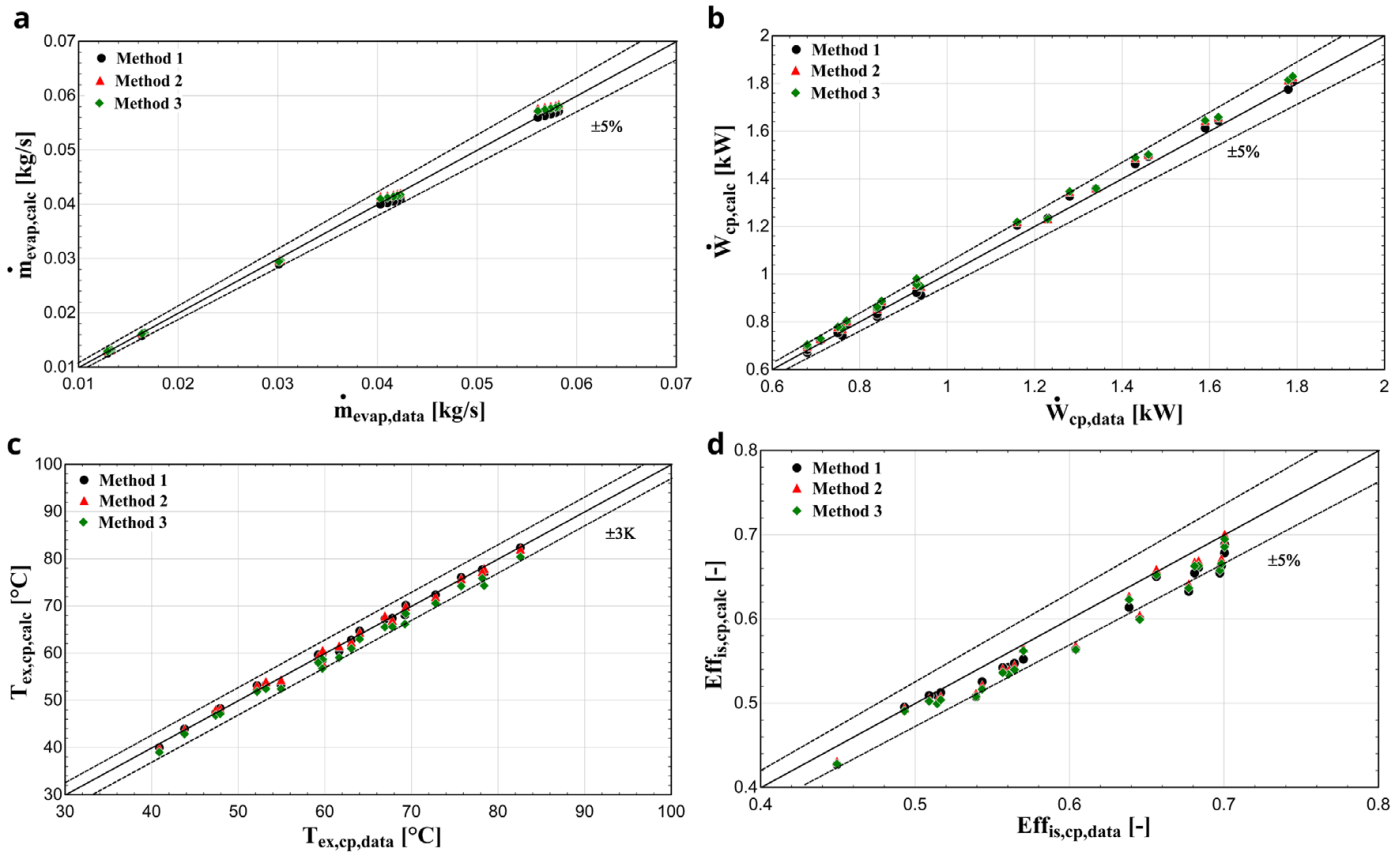
( **a**) Mass flow rate, ( **b**) electric power, ( **c**) discharge temperature, and ( **d**) isentropic efficiency comparison with ZR compressor and R450A for all three methods.

***Results with R450A.*** Comparing R450A with the reference refrigerant R134a, the saturation pressure distributions are almost identical ^31^ implying that similar performance characteristics are expected, as was the case of R134a with R1234yf for the YH compressor. This is very well demonstrated with the results of the three methods depicted in Figure 12.

The exhaust temperature, mass flow rate, and electric power predictions of all three methods are within the acceptable bandwidths verifying better model performance. The first two methods give very similar results, while the method 3 brings a slightly larger deviation, especially for the discharge temperature. On the other hand, the prediction of the isentropic efficiency is underestimated compared with the available data, with every method having a similar accuracy, with few conditions diverting by more than -5%.

***Overall accuracy of each method with the ZR compressor.*** Table 11 provides the average errors and standard deviations of the three methods for the refrigerant charge with the ZR compressor.

**Table 11.** Error analysis of the considered methodologies with ZR compressor.

		R407C			R1234yf		
		Method 1	Method 2	Method 3	Method 1	Method 2	Method 3
**Relative** **average** **error**	Discharge temperature (°C)	**1.07**	1.36	1.37	0.62	**0.60**	1.75
	Electric power (%)	**0.95**	2.41	2.40	**1.83**	3.02	3.01
	Mass flow rate (%)	0.97	**0.69**	2.09	2.49	**0.96**	1.14
	Isentropic efficiency (%)	**1.29**	2.49	2.43	3.47	**2.88**	3.58
**Standard** **deviation**	Discharge temperature (°C)	**0.69**	1.98	1.79	0.74	**0.71**	0.93
	Electric power (kW)	**0.02**	0.05	0.05	**0.02**	0.02	0.02
	Mass flow rate (kg/s)	0.00066	**0.00043**	0.00058	**0.00034**	0.00051	0.00044
	Isentropic efficiency (-)	**0.0045**	0.0169	0.0183	0.0139	**0.0125**	0.0126

For every case, the semi-empirical model provides reliable results with a good accuracy, especially with R450A. As it was also shown in the previous section, the least accurate method is the third one, showing systematically higher errors compared with the other two. However, its simplicity could favour its use in certain cases, which require the fast production of results and in case data are not available.

## Conclusions

Three methodologies have been developed and proposed based on a semi-empirical model for scroll compressors, with the first referring to the standard model with the complete set of parameters to be fine-tuned. In the second method, a reference refrigerant (R134a) is set for compressor-specific parameters, when only AUs and friction factor are identified by the optimization method, in order to examine the compressor performance for a different refrigerant. The third method is further simplified, since all parameters are calculated once for the reference refrigerant except from *AU _ex_* and *AU _su_*, which are identified by standard correlations for refrigerant change.

The prediction accuracy of the proposed semi empirical model was initially verified with a scroll compressor operating with R134a, showing deviations below 3.2 K for discharge temperature, 2.1% for mass flow rates and 4.3% for electric power compared with the manufacturer data. The next step was to apply the three methods to predict the same compressor performance, when operating with R407C and R1234yf. The accuracy of each method was then evaluated, with all methods predicting the discharge temperature within a ±3 K range with R407C. As expected, method 3 is the least accurate with the results exceeding the ±5% borderline for mass flow rate and electric power, reaching even a discrepancy of 10%. The accuracy is improved with R1234yf, due to its very similar thermophysical properties with the reference refrigerant.

To further verify the reliability of the methods and increase the confidence towards its wider applicability, the same procedure has been implemented with another scroll compressor operating with R134a, R407C and R450A, keeping the same reference refrigerant. For all three refrigerants, every method provides accurate results, keeping almost all operating conditions within the ±5% range for mass flow and electric power and ±3 K for discharge temperature.

The results presented in this work show that by generalizing the semi-empirical model for compressor simulation with different refrigerants (method 3) an acceptable prediction accuracy is reached, which is significantly improved when the reference refrigerant shows similar thermophysical characteristics to the simulated one. It is then expected that method 3 introduces greater deviations compared with methods 1 and 2, due to the model assumptions and lack of performance data for parameters fine-tuning, since the first two methods rely on the fine-tuning of the parameters for the specific pair compressor-refrigerant.

Finally, the outcome of this comparison is to provide an estimation of the semi-empirical model performance especially when no available data are available for parameters calibration, leading to method 3 as the only possible way to examine the compressor operation with a specific refrigerant. The discrepancies with all methods are acceptable for the purposes of such studies, leading to the conclusion that this approach is a reliable way of estimating the compressor and subsequently the overall system performance for different refrigerants. This becomes extremely important when investigating new refrigerants as drop-in replacements with existing compressors.

## Ethics and consent

None required.

## Data availability

### Underlying data

Zenodo: Compressor data with different refrigerants, https://doi.org/10.5281/zenodo.5702838

This project contains the following underlying data: Compressor semi-empirical model results with different refrigerants and three methods.xlsx (all data used to produce the results of this study). Data are available under the terms of the Creative Commons Zero “No rights reserved” data waiver (CC0 1.0 Public domain dedication).

## References

[1] Schulz M Kourkoulas D : Regulation (EU) No 517/2014 of The European Parliament and of the council of 16 April 2014 on fluorinated greenhouse gases and repealing Regulation (EC) No 842/2006. 2014. Reference Source

[2] Höglund-Isaksson L Purohit P Amann M : Cost estimates of the Kigali Amendment to phase-down hydrofluorocarbons. *Environ Sci Policy.* 2017;75:138–147. 10.1016/J.ENVSCI.2017.05.006

[3] Wu D Hu B Wang RZ : Vapor compression heat pumps with pure Low-GWP refrigerants. *Renew Sust Energ Rev.* 2021;138:110571. 10.1016/j.rser.2020.110571

[4] Mota-Babiloni A Mateu-Royo C Navarro-Esbrí J : Experimental comparison of HFO-1234ze(E) and R-515B to replace HFC-134a in heat pump water heaters and moderately high temperature heat pumps. *Appl Therm Eng.* 2021;196:117256. 10.1016/j.applthermaleng.2021.117256

[5] Fukuda S Kondou C Takata N : Low GWP refrigerants R1234ze(E) and R1234ze(Z) for high temperature heat pumps. *Int J Refrig.* 2014;40:161–173. 10.1016/J.IJREFRIG.2013.10.014

[6] Thu K Takezato K Takata N : Drop-in experiments and exergy assessment of HFC-32/HFO-1234yf/R744 mixture with GWP below 150 for domestic heat pumps. *Int J Refrig.* 2021;121:289–301. 10.1016/J.IJREFRIG.2020.10.009

[7] Sánchez D Cabello R Llopis R : Energy performance evaluation of R1234yf, R1234ze(E), R600a, R290 and R152a as low-GWP R134a alternatives. *Int J Refrig.* 2017;74:269–282. 10.1016/j.ijrefrig.2016.09.020

[8] Kosmadakis G Arpagaus C Neofytou P : Techno-economic analysis of high-temperature heat pumps with low-global warming potential refrigerants for upgrading waste heat up to 150 °C. *Energ Convers Manage.* 2020;226:113488. 10.1016/j.enconman.2020.113488

[9] Bell IH Ziviani D Lemort V : PDSim: A general quasi-steady modeling approach for positive displacement compressors and expanders. *Int J Refrig.* 2020;110:310–322. 10.1016/j.ijrefrig.2019.09.002

[10] Ziviani D Bell IH Zhang X : PDSim: Demonstrating the capabilities of an open-source simulation framework for positive displacement compressors and expanders. *Int J Refrig.* 2020;110:323–339. 10.1016/j.ijrefrig.2019.10.015

[11] Ma J Ding X Horton WT : Development of an automated compressor performance mapping using artificial neural network and multiple compressor technologies. *Int J Refrig.* 2020;120:66–80. 10.1016/j.ijrefrig.2020.08.001

[12] Duarte W Pabon JJG Maia AAT : Non-isentropic Phenomenological Model of a Reciprocating Compressor. *International Journal of Air-Conditioning and Refrigeration.* 2019;27(04):1950039. 10.1142/S2010132519500391

[13] Navarro E Granryd E Urchueguía JF : A phenomenological model for analyzing reciprocating compressors. *Int J Refrig.* 2007;30(7):1254–1265. 10.1016/j.ijrefrig.2007.02.006

[14] Yang B Bradshaw CR Groll EA : Modeling of a semi-hermetic CO _2_ reciprocating compressor including lubrication submodels for piston rings and bearings. *Int J Refrig.* 2013;36(7):1925–1937. 10.1016/j.ijrefrig.2012.10.017

[15] Ndiaye D Bernier M : Dynamic model of a hermetic reciprocating compressor in on–off cycling operation (Abbreviation: Compressor dynamic model). *Appl Therm Eng.* 2010;30(8–9):792–799. 10.1016/j.applthermaleng.2009.12.007

[16] Byrne P Ghoubali R Miriel J : Scroll compressor modelling for heat pumps using hydrocarbons as refrigerants. *Int J Refrig.* 2014;41:1–13. 10.1016/j.ijrefrig.2013.06.003

[17] Duprez ME Dumont E Frère M : Modeling of scroll compressors - Improvements. *Int J Refrig.* 2010;33(4):721–728. 10.1016/j.ijrefrig.2009.12.025

[18] Mateu-Royo C Mota-Babiloni A Navarro-Esbrí J : Semi-empirical and environmental assessment of the low GWP refrigerant HCFO-1224yd(Z) to replace HFC-245fa in high temperature heat pumps. *Int J Refrig.* 2021;127:120–127. 10.1016/j.ijrefrig.2021.02.018

[19] Muye J Praveen Kumar G Bruno JC : Modelling of scroll expander for different working fluids for low capacity power generation. *Appl Therm Eng.* 2019;159:113932. 10.1016/j.applthermaleng.2019.113932

[20] Winandy E Saavedra OC Lebrun J : Experimental analysis and simplified modelling of a hermetic scroll refrigeration compressor. *Appl Therm Eng.* 2002;22(2):107–120. 10.1016/S1359-4311(01)00083-7

[21] Dardenne L Fraccari E Maggioni A : Semi-empirical modelling of a variable speed scroll compressor with vapour injection. *Int J Refrig.* 2015;54:76–87. 10.1016/j.ijrefrig.2015.03.004

[22] Cuevas C Lebrun J Lemort V : Characterization of a scroll compressor under extended operating conditions. *Appl Therm Eng.* 2010;30(6–7):605–615. 10.1016/j.applthermaleng.2009.11.005

[23] D’Amico F Pallis P Leontaritis AD : Semi-empirical model of a multi-diaphragm pump in an Organic Rankine Cycle (ORC) experimental unit. *Energy.* 2018;143:1056–1071. 10.1016/j.energy.2017.10.127

[24] Tello-Oquendo FM Navarro-Peris E Barceló-Ruescas F : Semi-empirical model of scroll compressors and its extension to describe vapor-injection compressors. Model description and experimental validation. *Int J Refrig.* 2019;106:308–326. 10.1016/j.ijrefrig.2019.06.031

[25] Cuevas C Lebrun J : Testing and modelling of a variable speed scroll compressor. *Appl Therm Eng.* 2009;29(2–3):469–478. 10.1016/j.applthermaleng.2008.03.016

[26] Klein SA : Engineering Equation Solver-EES (64-bit). 2020. Reference Source

[27] Giuffrida A : Modelling the performance of a scroll expander for small organic Rankine cycles when changing the working fluid. *Appl Therm Eng.* 2014;70(1):1040–1049. 10.1016/j.applthermaleng.2014.06.004

[28] Giuffrida A : Improving the semi-empirical modelling of a single-screw expander for small organic Rankine cycles. *Appl Energ.* 2017;193:356–368. 10.1016/j.apenergy.2017.02.015

[29] Reasor P Aute V Radermacher R : Refrigerant R1234yf Performance Comparison Investigation. *International Refrigeration and Air Conditioning Conference.* 2010;1085. Reference Source

[30] Vaghela JK : Comparative Evaluation of an Automobile Air - Conditioning System Using R134a and Its Alternative Refrigerants. *Enrgy Proced.* 2017;109:153–160. 10.1016/j.egypro.2017.03.083

[31] Makhnatch P Mota-Babiloni A López-Belchí A : R450A and R513A as lower GWP mixtures for high ambient temperature countries: Experimental comparison with R134a. *Energy.* 2019;166:223–235. 10.1016/j.energy.2018.09.001

