# Supplementary material for: Methods based on a semi-empirical model for simulating scroll compressors with HFC and HFO refrigerants
Source: Open Res Eur. 2022 Jul 7;1:148. Originally published 2021 Dec 8. [Version 3] doi: 10.12688/openreseurope.14313.3 (PMC10446056; doi:10.12688/openreseurope.14313.3)
Supplement: Peer review history

# Peer review: Reviewer response for version 3

**DOI:** 10.21956/openreseurope.16145.r29655

**Published:** 2022-07-26

**Authors:** Stefano Barberis

**Affiliations:** 1 TGP - DIME - Department of Mechanical Engineering, University of Genoa, Genoa, Italy

The authors have adequately addressed my concerns. Just a minor suggestion - as this is an article coming from an EU funded project, it could make sense to mention other EU funded projects and their related papers working on similar topics.

Is the rationale for developing the new method (or application) clearly explained?

Partly

Is the description of the method technically sound?

Yes

Are the conclusions about the method and its performance adequately supported by the findings presented in the article?

Yes

If any results are presented, are all the source data underlying the results available to ensure full reproducibility?

Yes

Are sufficient details provided to allow replication of the method development and its use by others?

Yes

Reviewer Expertise:

Energy systems

I confirm that I have read this submission and believe that I have an appropriate level of expertise to confirm that it is of an acceptable scientific standard.

# Peer review: Reviewer response for version 3

**DOI:** 10.21956/openreseurope.16145.r29654

**Published:** 2022-07-07

**Authors:** Adrián Mota-Babiloni

**Affiliations:** 1 ISTENER Research Group, Department of Mechanical Engineering and Construction, Universitat Jaume I, Castellón de la Plana, Spain

The authors have improved and modified the paper accordingly.

Is the rationale for developing the new method (or application) clearly explained?

Yes

Is the description of the method technically sound?

Yes

Are the conclusions about the method and its performance adequately supported by the findings presented in the article?

Yes

If any results are presented, are all the source data underlying the results available to ensure full reproducibility?

Yes

Are sufficient details provided to allow replication of the method development and its use by others?

Yes

Reviewer Expertise:

refrigeration, applied thermodynamics, heat pumps

I confirm that I have read this submission and believe that I have an appropriate level of expertise to confirm that it is of an acceptable scientific standard.

# Peer review: Reviewer response for version 2

**DOI:** 10.21956/openreseurope.15821.r29281

**Published:** 2022-05-27

**Authors:** Adrián Mota-Babiloni

**Affiliations:** 1 ISTENER Research Group, Department of Mechanical Engineering and Construction, Universitat Jaume I, Castellón de la Plana, Spain

This paper presents a comparison of models for scroll compressor behaviour prediction. The topic is interesting and novel. The methodology is adequate and well explained. I recommend minor modifications as follows:

1. Nomenclature: Coefficient of Performance should be a parameter, not an abbreviation.
2. Introduction F-Gas regulation --> F-gas Regulation, Kigali amendment --> Kigali Amendment.
3.  Introduction: Be careful with this sentence "Attention is also given on refrigerant mixtures (e.g. HFC with HFO blends) due to their wide flexibility in thermodynamic and GWP characteristics, since pure substances are not so easily adapted". I recommend rewriting it to be more accurate.
4. When referencing researchers with two surnames in the text (mostly Spanish or Latin-American), include both surnames with a dash in between.
5. When mentioning hydrocarbons, you can call them by the refrigerant designation (R600a, R290) or name them (Isobutane, Propane). Choose only one way.
6.  Include References in Figure captions (besides text) if they are extracted from other sources (for instance, Fig 1 and 2).
7. Figure 3. Superheating instead of superheat (because you already have subcooling). Then, power should be electric power, and suction mass flow should be suction mass flow rate.
8. For all figures comparing experimental and predicted data, produce Figures and graphics squared, not rectangular. If Graphics are a square, it is easier to check a deviation (this is mandatory in some journals such as JIJR).
9. Justify why you selected R407C, R1234yf and R450A. Usually, researchers classify refrigerants (many properties depend on this) depending on the molecular mass. Have you considered this?
10. Comment in detail differences in prediction methods for different compressors and refrigerants.
11. Use the symbol for efficiency eta instead of Eff.
12. Standard deviation shown in tables should have the plus-minus sign?
13. Table 10 requires units.

After these modifications are applied, the paper can be approved.

Is the rationale for developing the new method (or application) clearly explained?

Yes

Is the description of the method technically sound?

Yes

Are the conclusions about the method and its performance adequately supported by the findings presented in the article?

Yes

If any results are presented, are all the source data underlying the results available to ensure full reproducibility?

Yes

Are sufficient details provided to allow replication of the method development and its use by others?

Yes

Reviewer Expertise:

refrigeration, applied thermodynamics, heat pumps

I confirm that I have read this submission and believe that I have an appropriate level of expertise to confirm that it is of an acceptable scientific standard, however I have significant reservations, as outlined above.

# Response

**Published:** 2022-06-26

**Authors:** George Kosmadakis

**Affiliations:** National Center for Scientific Research Demokritos, Greece

The authors would like to thank the reviewer for his valuable comments that greatly helped for improving this work. All comments have been addressed, providing detailed answers to each comment separately and indicating any change that has been made in the revised manuscript.

**Reviewer #3**:

*Reviewer:* This paper presents a comparison of models for scroll compressor behaviour prediction. The topic is interesting and novel. The methodology is adequate and well explained. I recommend minor modifications as follows: Nomenclature: Coefficient of Performance should be a parameter, not an abbreviation.  

*Authors*: The suggested change has been implemented.

*Reviewer:* Introduction F-Gas regulation --> F-gas Regulation, Kigali amendment --> Kigali Amendment.

*Authors*: This part of the introduction has been revised.

*Reviewer:* Introduction: Be careful with this sentence "Attention is also given on refrigerant mixtures (e.g. HFC with HFO blends) due to their wide flexibility in thermodynamic and GWP characteristics, since pure substances are not so easily adapted". I recommend rewriting it to be more accurate.

*Authors*: The suggested change has been implemented.

*Reviewer:* When referencing researchers with two surnames in the text (mostly Spanish or Latin-American), include both surnames with a dash in between.

*Authors*: The suggested change has been implemented.

*Reviewer:* When mentioning hydrocarbons, you can call them by the refrigerant designation (R600a, R290) or name them (Isobutane, Propane). Choose only one way.

*Authors*: The suggested change has been implemented (e.g. in Table 1).

*Reviewer:* Include References in Figure captions (besides text) if they are extracted from other sources (for instance, Fig 1 and 2).

*Authors*: All the figures (including Figs. 1 and 2) have been created by the authors. Therefore, there is no need to add any references.

*Reviewer:* Figure 3. Superheating instead of superheat (because you already have subcooling). Then, power should be electric power, and suction mass flow should be suction mass flow rate.

*Authors*: The suggested change has been implemented.

*Reviewer:* For all figures comparing experimental and predicted data, produce Figures and graphics squared, not rectangular. If Graphics are a square, it is easier to check a deviation (this is mandatory in some journals such as JIJR).

*Authors*: The suggested change has been implemented.

*Reviewer:* Justify why you selected R407C, R1234yf and R450A. Usually, researchers classify refrigerants (many properties depend on this) depending on the molecular mass. Have you considered this?

*Authors*: The selection criteria of the refrigerants did not include their molecular mass. Instead, as also replied to the 2 ^nd^ reviewer, the main focus was to select refrigerants that cover a very large range of GWP, from 4 up to almost 2000. Moreover, R134a and R407C are selected since they are well established refrigerants which are available for both compressor models in order to facilitate the comparison.

*Reviewer:* Comment in detail differences in prediction methods for different compressors and refrigerants.

*Authors*: More details about the differences in prediction methods have been added after Table 9 (for the YH compressor) and Table 11 (for the ZR compressor).

*Reviewer:* Use the symbol for efficiency eta instead of Eff.

*Authors*: The suggested change has been implemented.

*Reviewer:* Standard deviation shown in tables should have the plus-minus sign?

*Authors*: The provided deviation in Table 7 is not the standard one but the minimum – maximum deviation between the calculated and target values. In the other tables (e.g. Tables 9 and 11) where standard deviation is given, the values have no signs.

*Reviewer:* Table 10 requires units.

*Authors* : The suggested change has been implemented.

# Peer review: Reviewer response for version 2

**DOI:** 10.21956/openreseurope.15821.r29282

**Published:** 2022-05-27

**Authors:** Stefano Barberis

**Affiliations:** 1 TGP - DIME - Department of Mechanical Engineering, University of Genoa, Genoa, Italy

The goal of this paper is to present simplified models to simulate the performances of scroll compressors (to be applied we assume in HPs and refrigeration systems even if not fully described in the paper) in order to compare the performances of the compressors if operating with different refrigerants and therefore targeting the identification of the most suitable ones.

 Generally speaking, the paper is easy to read and sound, nevertheless some minor adjustments are suggested:

1. In the introduction, please include a better explanation of the R&D context (e.g. what is the role of this activity in RES4BUILD, why has this model has been developed, for which general-purpose and project-specific purpose?) and of research objectives (e.g. study innovative/low GWP refrigerants?).
2. Please explain why you choose the analysed refrigerants.
3. In equation 10 you state: *Hence, a power exponent of 0.8 is typically used according to the Reynolds analogy in a fully developed turbulent flow. *Please add a reference that enabled you to identify this 0.8 value.

Is the rationale for developing the new method (or application) clearly explained?

Partly

Is the description of the method technically sound?

Yes

Are the conclusions about the method and its performance adequately supported by the findings presented in the article?

Yes

If any results are presented, are all the source data underlying the results available to ensure full reproducibility?

Yes

Are sufficient details provided to allow replication of the method development and its use by others?

Yes

Reviewer Expertise:

Energy systems

I confirm that I have read this submission and believe that I have an appropriate level of expertise to confirm that it is of an acceptable scientific standard, however I have significant reservations, as outlined above.

# Response

**Published:** 2022-06-26

**Authors:** George Kosmadakis

**Affiliations:** National Center for Scientific Research Demokritos, Greece

The authors would like to thank the Reviewer for his valuable comments that greatly helped for improving this work. All comments have been addressed, providing detailed answers to each comment separately and indicating any change that has been made in the revised manuscript.

**Reviewer #2**:

*Reviewer:* The goal of this paper is to present simplified models to simulate the performances of scroll compressors (to be applied we assume in HPs and refrigeration systems even if not fully described in the paper) in order to compare the performances of the compressors if operating with different refrigerants and therefore targeting the identification of the most suitable ones. Generally speaking, the paper is easy to read and sound, nevertheless some minor adjustments are suggested: In the introduction, please include a better explanation of the R&D context (e.g. what is the role of this activity in RES4BUILD, why has this model has been developed, for which general-purpose and project-specific purpose?) and of research objectives (e.g. study innovative/low GWP refrigerants?).

*Authors*: The general and specific purpose of this work has been added in the last paragraph of Introduction.

*Reviewer:* Please explain why you choose the analysed refrigerants.

*Authors*: One of the aims of this work is to analyse refrigerants with ultra-low up to high GWP. This is why the specific refrigerants have been selected (not an exhaustive list though). This has been further clarified in the revised manuscript by enriching the text between Tables 3 and 4.

*Reviewer:* In equation 10 you state: Hence, a power exponent of 0.8 is typically used according to the Reynolds analogy in a fully developed turbulent flow. Please add a reference that enabled you to identify this 0.8 value.

*Authors*: A reference for this statement has been added.

# Peer review: Reviewer response for version 2

**DOI:** 10.21956/openreseurope.15821.r28930

**Published:** 2022-03-28

**Authors:** Willian Moreira Duarte

**Affiliations:** 1 Postgraduate Program in Mechanical Engineering, Federal University of Minas Gerais (UFMG), Belo Horizonte, Brazil

All issues have been addressed by the authors.

Is the rationale for developing the new method (or application) clearly explained?

Yes

Is the description of the method technically sound?

Yes

Are the conclusions about the method and its performance adequately supported by the findings presented in the article?

Yes

If any results are presented, are all the source data underlying the results available to ensure full reproducibility?

Yes

Are sufficient details provided to allow replication of the method development and its use by others?

Yes

Reviewer Expertise:

NA

I confirm that I have read this submission and believe that I have an appropriate level of expertise to confirm that it is of an acceptable scientific standard.

# Peer review: Reviewer response for version 1

**DOI:** 10.21956/openreseurope.15442.r28276

**Published:** 2022-01-25

**Authors:** Willian Moreira Duarte

**Affiliations:** 1 Postgraduate Program in Mechanical Engineering, Federal University of Minas Gerais (UFMG), Belo Horizonte, Brazil

The article presents a phenomenological model for the thermodynamic processes of a compressor of a refrigeration system operating at steady state. The model is based on the application of the energy equations in the pre-compression, compression, and post-compression process. Three methods are proposed for calibration of the model. The methods are validated using the compressor manufacture data. The model was used to compare different refrigerants. The manuscript was found to be relevant to the aim and scope of the journal.

 However, some issues still need to be improved:

  

- Check equation 24. The equation 24 in the way presented is not dimensionless. The reviewer thinks that the mass flow rate is missing in the numerator.
- Q (in Figure 1) is not defined, only .
- The letter N was used for the rotation speed and the number of data (Eq. 4 and 25).
- The authors should present a table comparing the compressor models available in the literature. For this table the reviewer suggests expanding the literature review considering the following articles:
  - https://doi.org/10.1016/j.ijrefrig.2019.09.002
  - https://doi.org/10.1016/j.ijrefrig.2019.10.015
  - https://doi.org/10.1016/j.ijrefrig.2020.08.001
  - https://doi.org/10.1142/S2010132519500391
  - https://doi.org/10.1016/j.applthermaleng.2009.12.007
  - http://dx.doi.org/10.1016/j.ijrefrig.2012.10.017
  - https://doi.org/10.1016/j.ijrefrig.2007.02.006

Is the rationale for developing the new method (or application) clearly explained?

Yes

Is the description of the method technically sound?

Yes

Are the conclusions about the method and its performance adequately supported by the findings presented in the article?

Yes

If any results are presented, are all the source data underlying the results available to ensure full reproducibility?

Yes

Are sufficient details provided to allow replication of the method development and its use by others?

Yes

Reviewer Expertise:

NA

I confirm that I have read this submission and believe that I have an appropriate level of expertise to confirm that it is of an acceptable scientific standard, however I have significant reservations, as outlined above.

## References

[1] : PDSim: A general quasi-steady modeling approach for positive displacement compressors and expanders. *International Journal of Refrigeration* .2020;110: 10.1016/j.ijrefrig.2019.09.002 310-322 10.1016/j.ijrefrig.2019.09.002

[2] : PDSim: Demonstrating the capabilities of an open-source simulation framework for positive displacement compressors and expanders. *International Journal of Refrigeration* .2020;110: 10.1016/j.ijrefrig.2019.10.015 323-339 10.1016/j.ijrefrig.2019.10.015

[3] : Development of an automated compressor performance mapping using artificial neural network and multiple compressor technologies. *International Journal of Refrigeration* .2020;120: 10.1016/j.ijrefrig.2020.08.001 66-80 10.1016/j.ijrefrig.2020.08.001

[4] : Nonisentropic Phenomenological Model of a Reciprocating Compressor. *International Journal of Air-Conditioning and Refrigeration* .2019;27(04) : 10.1142/S2010132519500391 10.1142/S2010132519500391

[5] : Dynamic model of a hermetic reciprocating compressor in on–off cycling operation (Abbreviation: Compressor dynamic model). *Applied Thermal Engineering* .2010;30(8-9) : 10.1016/j.applthermaleng.2009.12.007 792-799 10.1016/j.applthermaleng.2009.12.007

[6] : Modeling of a semi-hermetic CO2 reciprocating compressor including lubrication submodels for piston rings and bearings. *International Journal of Refrigeration* .2013;36(7) : 10.1016/j.ijrefrig.2012.10.017 1925-1937 10.1016/j.ijrefrig.2012.10.017

[7] : A phenomenological model for analyzing reciprocating compressors. *International Journal of Refrigeration* .2007;30(7) : 10.1016/j.ijrefrig.2007.02.006 1254-1265 10.1016/j.ijrefrig.2007.02.006

# Response

**Published:** 2022-03-17

**Authors:** George Kosmadakis

**Affiliations:** National Center for Scientific Research Demokritos, Greece

The authors would like to thank the Reviewer for his valuable comments that greatly helped in improving this work. All comments have been addressed, providing detailed answers to each comment separately and indicating any change that has been made in the revised manuscript.

**Reviewer #1**: *Reviewer:* Check equation 24. The equation 24 in the way presented is not dimensionless. The reviewer thinks that the mass flow rate is missing in the numerator. *Authors*: We would like to thank the reviewer for pointing this out. The mass flow rate has been added to the numerator of Eq. (24).  

*Reviewer:* Q (in Figure 1) is not defined, only  .

*Authors*: Figure 1 has been revised. Q has been replaced by *Q*  showing the energy flow in the compressor sub-processes.  

*Reviewer:* The letter N was used for the rotation speed and the number of data (Eq. 4 and 25). *Authors*: The symbol of the number of data has been replaced with Z in order to differentiate it from the rotational speed. The symbol Z has also been added to the Nomenclature table.  

*Reviewer:* The authors should present a table comparing the compressor models available in the literature. For this table the reviewer suggests expanding the literature review considering the following articles:

-
*https://doi.org/10.1016/j.ijrefrig.2019.09.002*

-
*https://doi.org/10.1016/j.ijrefrig.2019.10.015*

-
*https://doi.org/10.1016/j.ijrefrig.2020.08.001*

- *https://doi.org/10.1142/S2010132519500391*
- *https://doi.org/10.1016/j.applthermaleng.2009.12.007*
- *http://dx.doi.org/10.1016/j.ijrefrig.2012.10.017*
- *https://doi.org/10.1016/j.ijrefrig.2007.02.006*

 
*Authors*: All the suggested scientific papers have been taken into consideration and added to the literature review in the Introduction, included in a new Table (Table 1).
